# Supplementary figures and images for: FBXL4 ubiquitin ligase deficiency promotes mitophagy by elevating NIX levels
Source: EMBO J. 2023 Apr 27;42(13):e112799. doi: 10.15252/embj.2022112799 (PMC10308357; doi:10.15252/embj.2022112799)

Ctrl1 KO1 Ctrl2 KO2

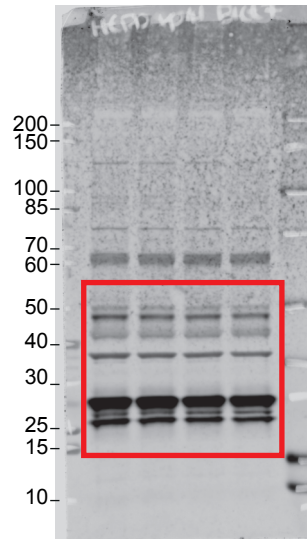

Cathepsin D-680

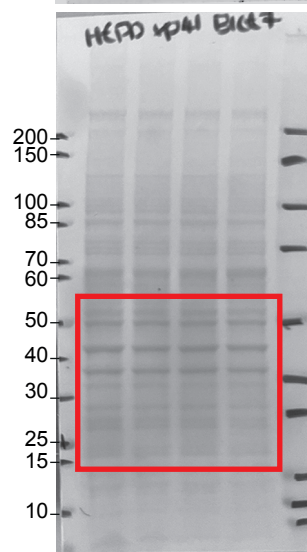

Ponceau

Supplement: Supplementary file 2 — Source Data for Expanded View [file EMBJ-42-e112799-s011.zip › EMBOJ-2022-112799R_ExpandedViewSourceData/EMBOJ-2022-112799R_EV2/Figure EV2F/EMBOJ-2022-112799R-Figure_EV2F_Source_Data-sd.pdf]

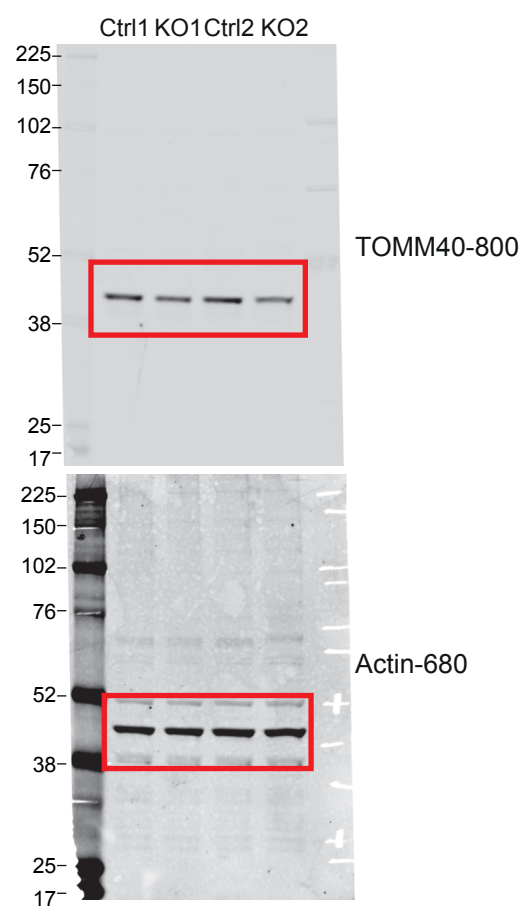

Supplement: Supplementary file 2 — Source Data for Expanded View [file EMBJ-42-e112799-s011.zip › EMBOJ-2022-112799R_ExpandedViewSourceData/EMBOJ-2022-112799R_EV2/Figure EV2D/EMBOJ-2022-112799R-Figure_EV2D_Source_Data-sd.pdf]

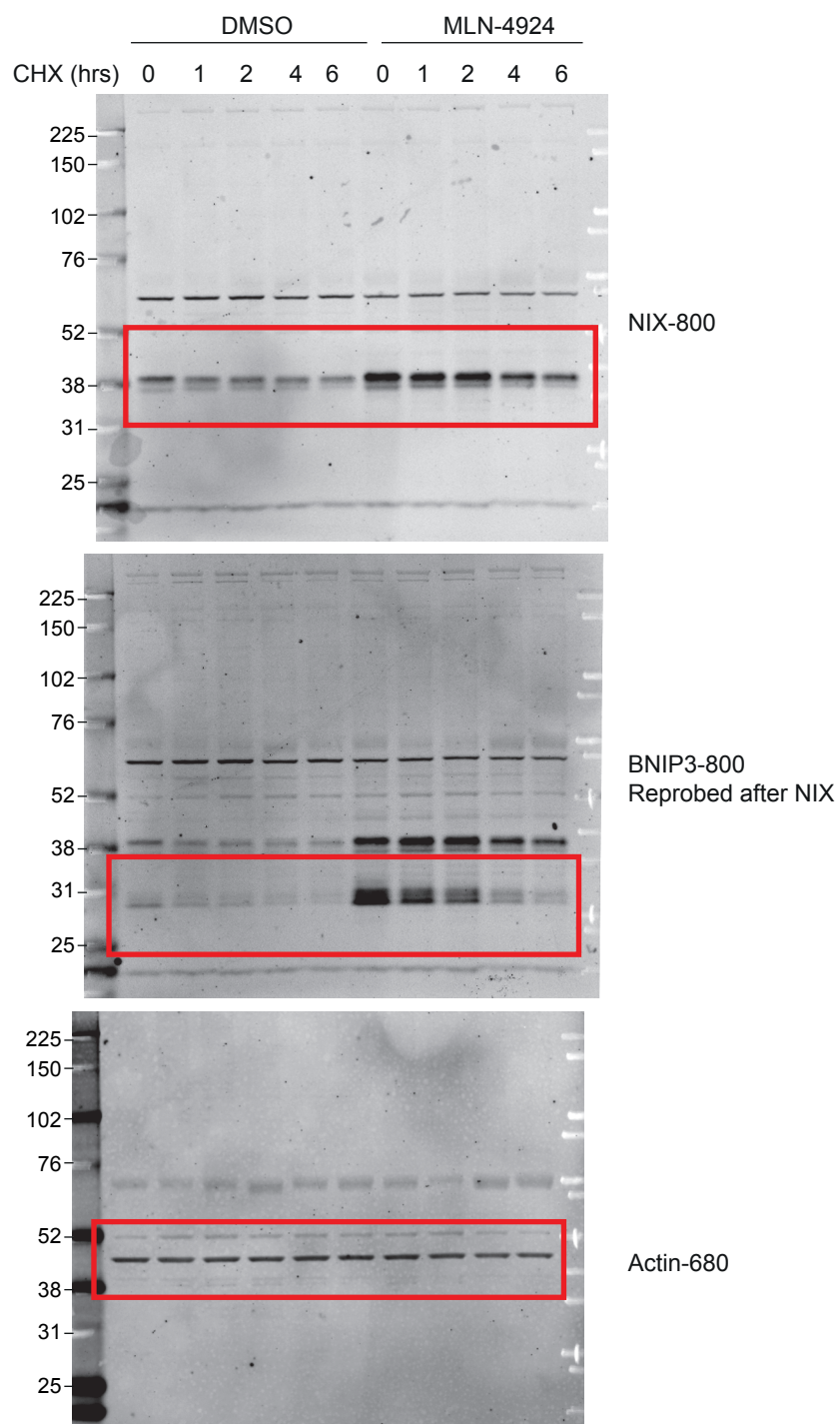

Supplement: Supplementary file 2 — Source Data for Expanded View [file EMBJ-42-e112799-s011.zip › EMBOJ-2022-112799R_ExpandedViewSourceData/EMBOJ-2022-112799R_EV3/Figure EV3D/Figure EV3D-Western blot.pdf]

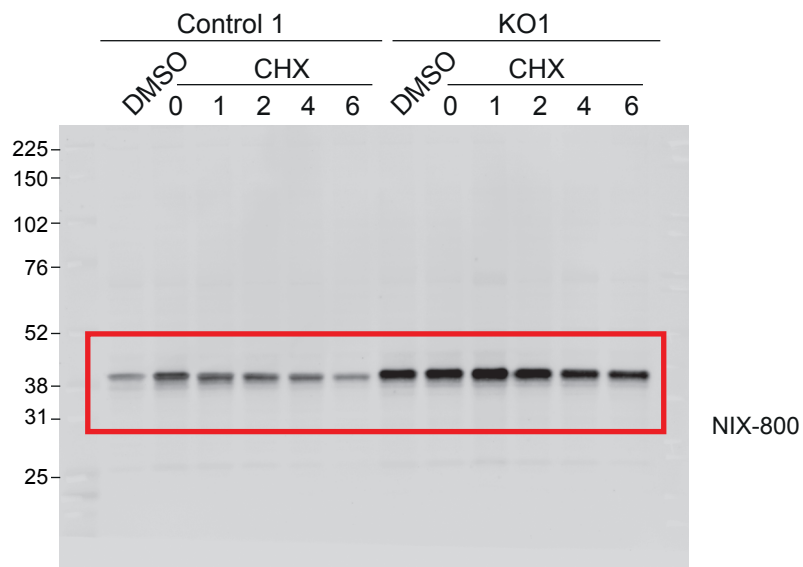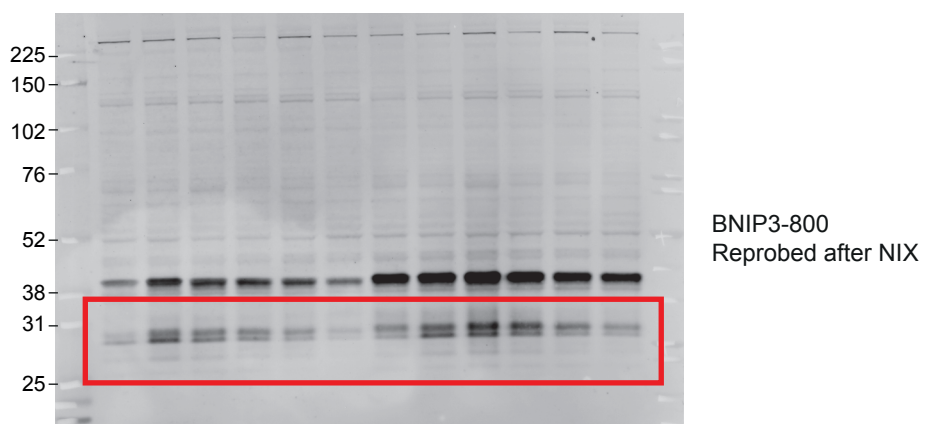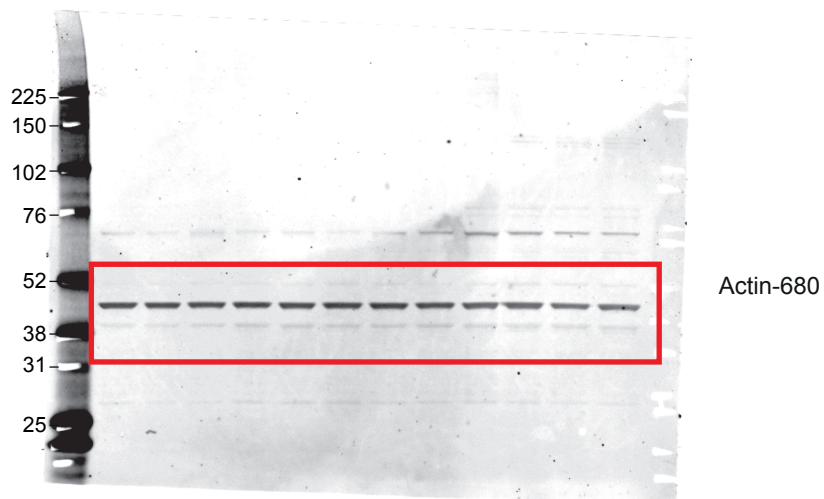

Supplement: Supplementary file 2 — Source Data for Expanded View [file EMBJ-42-e112799-s011.zip › EMBOJ-2022-112799R_ExpandedViewSourceData/EMBOJ-2022-112799R_EV3/Figure EV3E/Figure EV3E-Western blot.pdf]

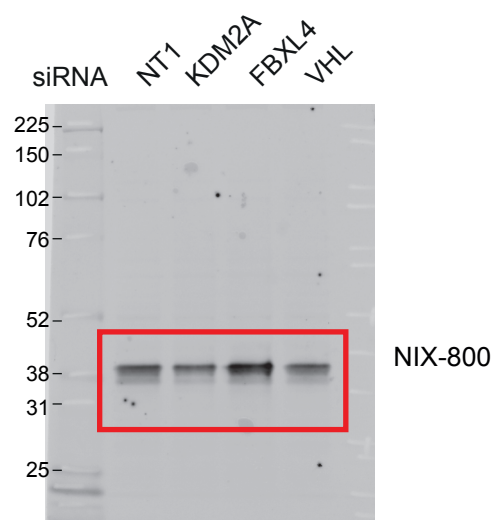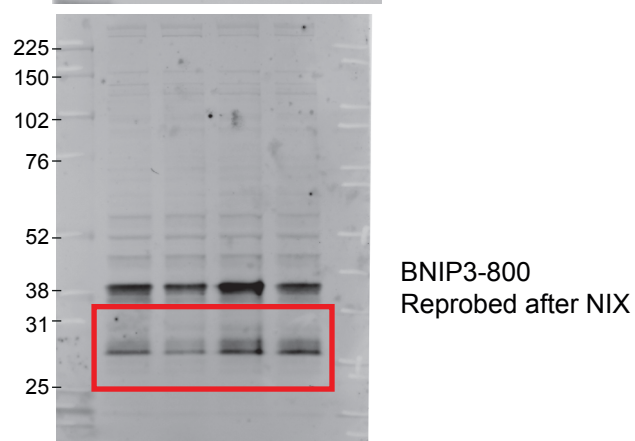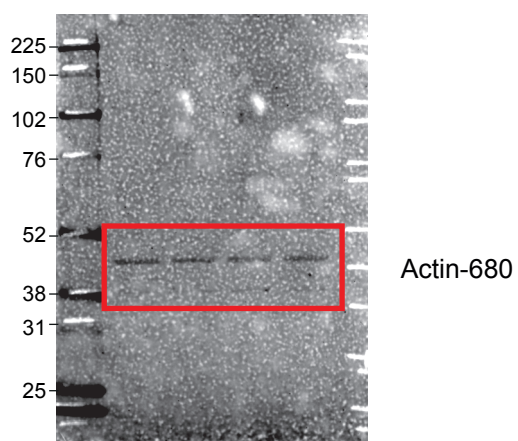

Supplement: Supplementary file 2 — Source Data for Expanded View [file EMBJ-42-e112799-s011.zip › EMBOJ-2022-112799R_ExpandedViewSourceData/EMBOJ-2022-112799R_EV3/Figure EV3H/Figure EV3H-Western blot.pdf]

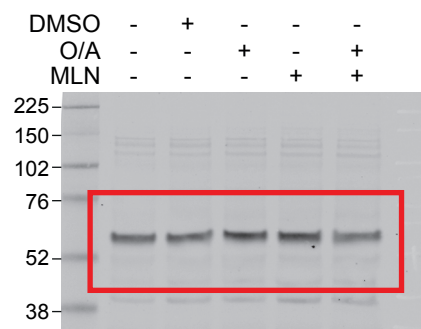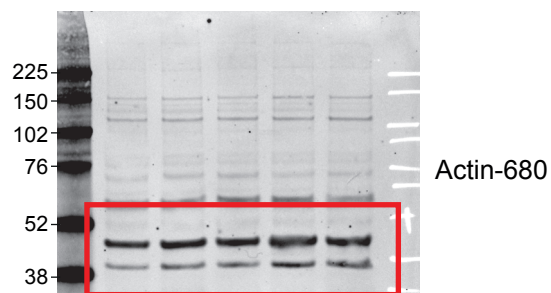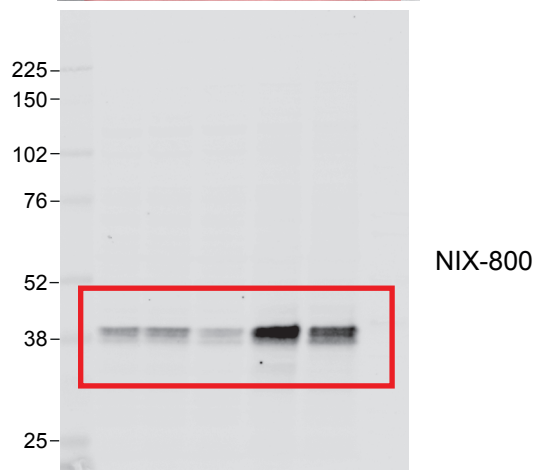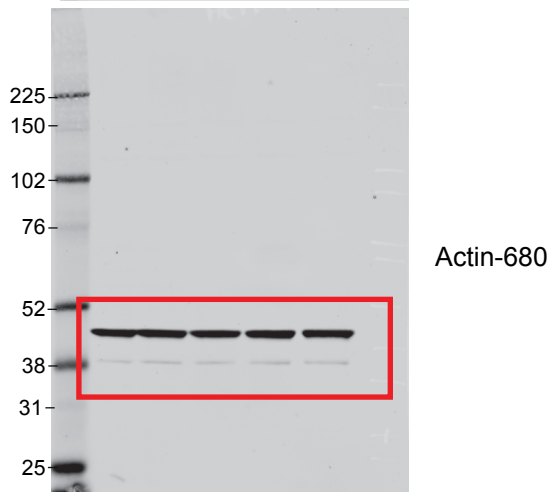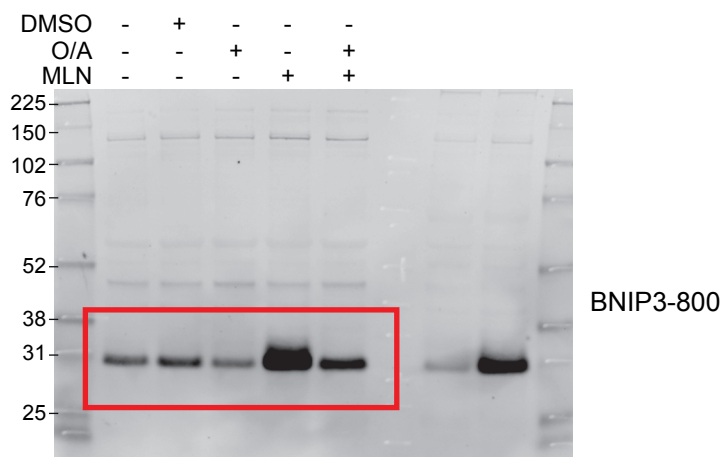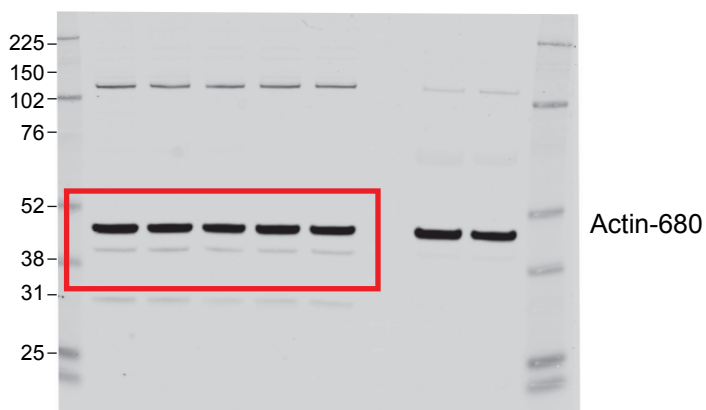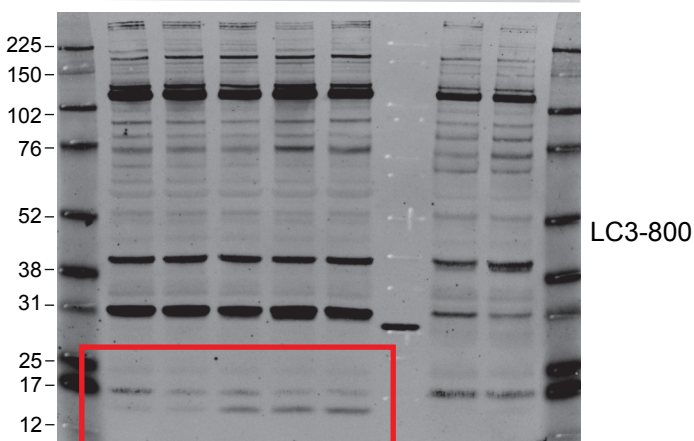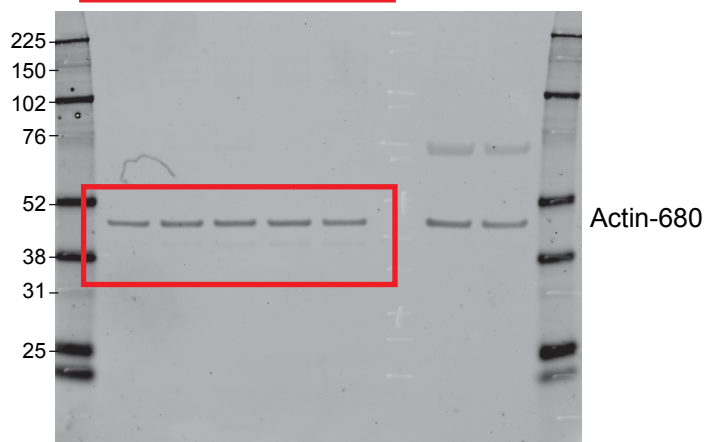

Supplement: Supplementary file 2 — Source Data for Expanded View [file EMBJ-42-e112799-s011.zip › EMBOJ-2022-112799R_ExpandedViewSourceData/EMBOJ-2022-112799R_EV3/Figure EV3A/Figure EV3A-Western blot.pdf]

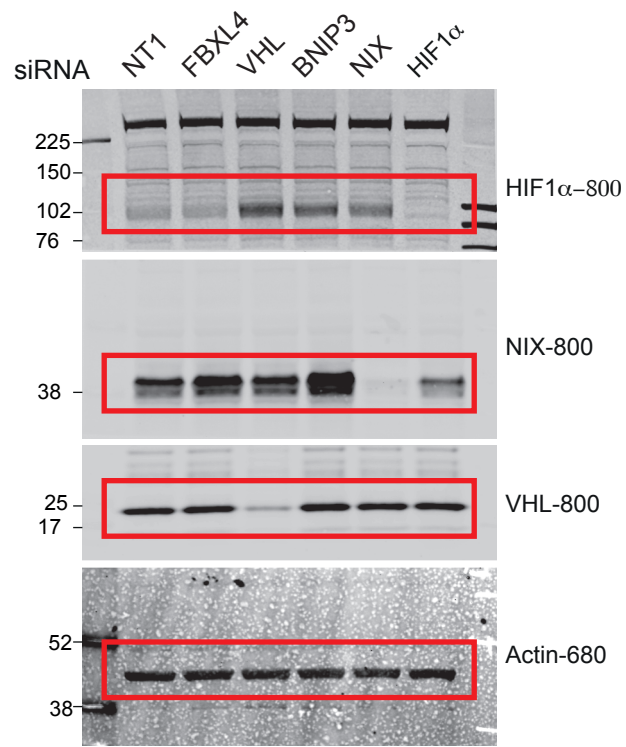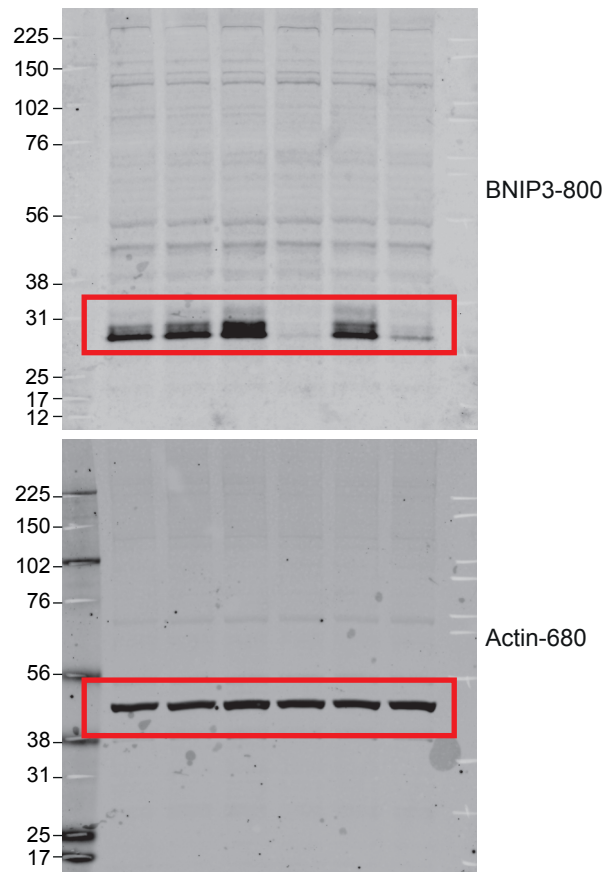

Supplement: Supplementary file 6 — Source Data for Figure 2 [file EMBJ-42-e112799-s001.zip › EMBOJ-2022-112799R_Figure2/Figure 2A/Figure 2A-Western blot.pdf]

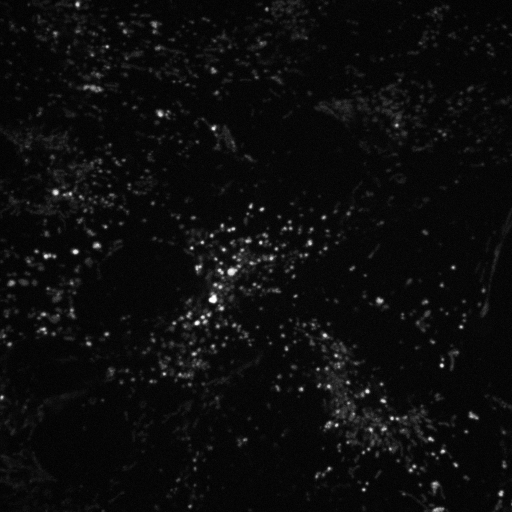

Supplement: Supplementary file 7 — Source Data for Figure 3 [file EMBJ-42-e112799-s009.zip › EMBOJ-2022-112799R_Figure3/Figure 3D/KO1 Ex. 561.tif]

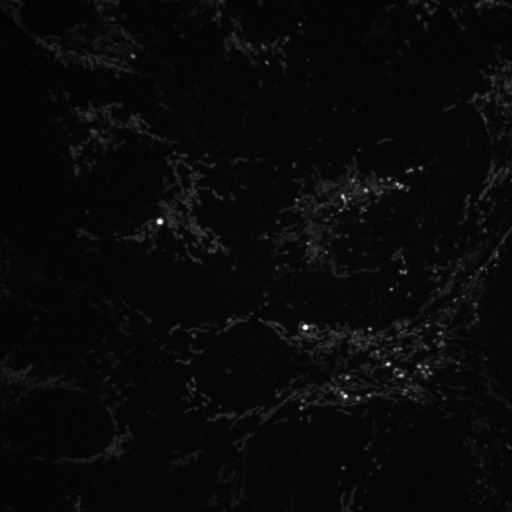

Supplement: Supplementary file 7 — Source Data for Figure 3 [file EMBJ-42-e112799-s009.zip › EMBOJ-2022-112799R_Figure3/Figure 3D/Control 2 Ex.561.tif]

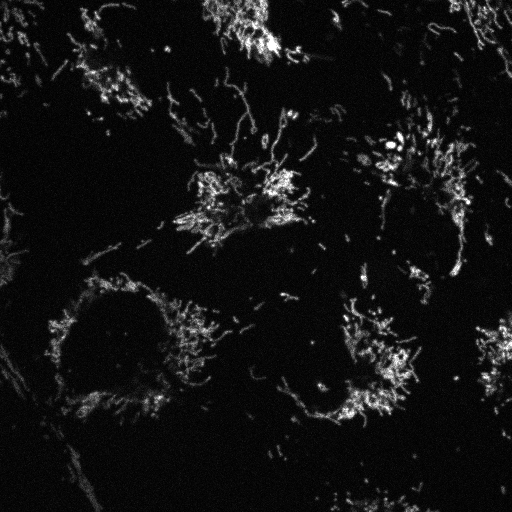

Supplement: Supplementary file 7 — Source Data for Figure 3 [file EMBJ-42-e112799-s009.zip › EMBOJ-2022-112799R_Figure3/Figure 3D/Control 1 Ex.455.tif]

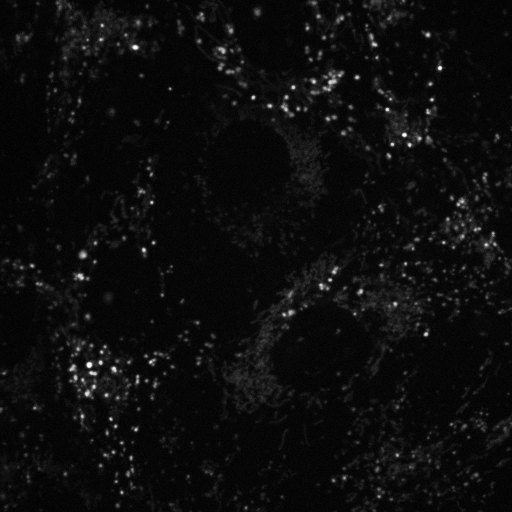

Supplement: Supplementary file 7 — Source Data for Figure 3 [file EMBJ-42-e112799-s009.zip › EMBOJ-2022-112799R_Figure3/Figure 3D/KO2 Ex. 561.tif]

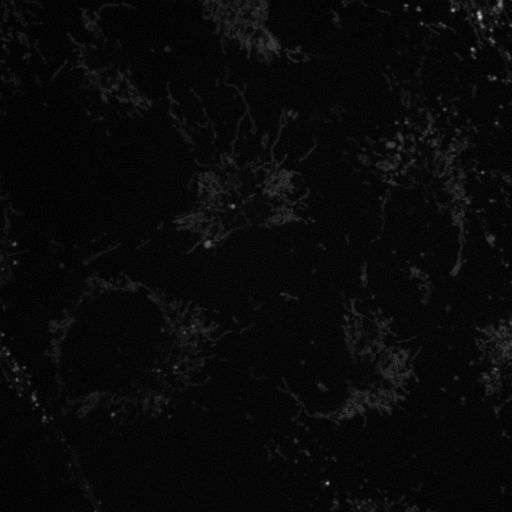

Supplement: Supplementary file 7 — Source Data for Figure 3 [file EMBJ-42-e112799-s009.zip › EMBOJ-2022-112799R_Figure3/Figure 3D/Control 1 Ex. 561.tif]

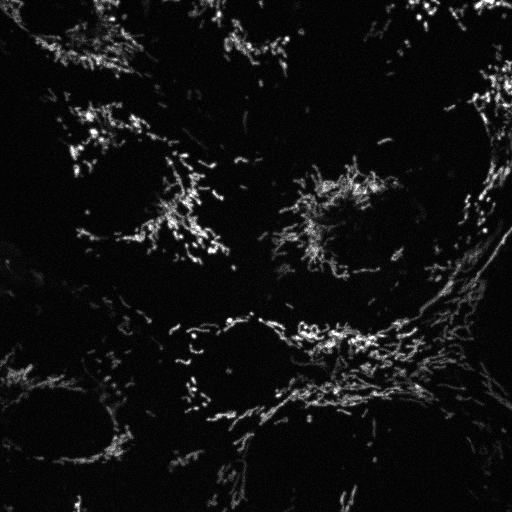

Supplement: Supplementary file 7 — Source Data for Figure 3 [file EMBJ-42-e112799-s009.zip › EMBOJ-2022-112799R_Figure3/Figure 3D/Control 2 Ex.445.tif]

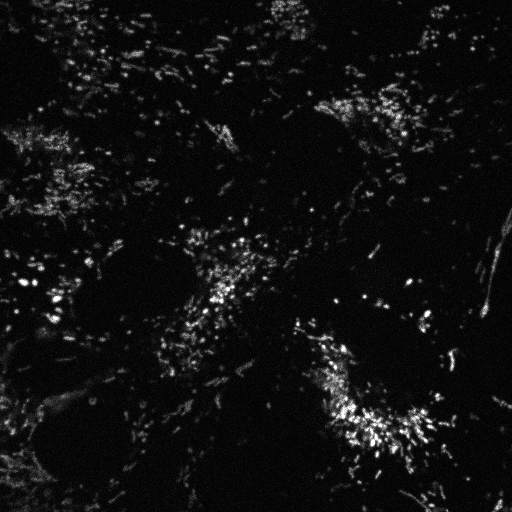

Supplement: Supplementary file 7 — Source Data for Figure 3 [file EMBJ-42-e112799-s009.zip › EMBOJ-2022-112799R_Figure3/Figure 3D/KO1 Ex. 445.tif]

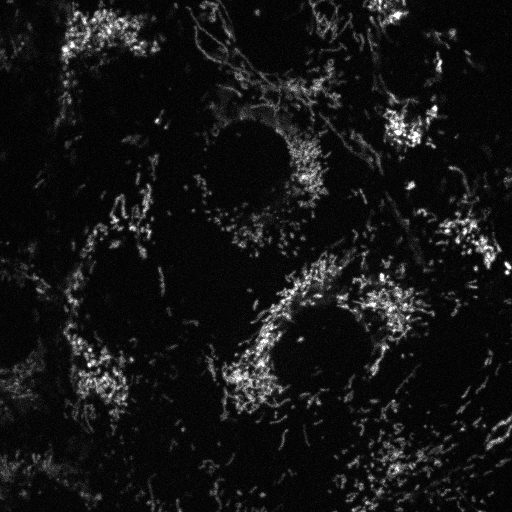

Supplement: Supplementary file 7 — Source Data for Figure 3 [file EMBJ-42-e112799-s009.zip › EMBOJ-2022-112799R_Figure3/Figure 3D/KO2 Ex. 445.tif]

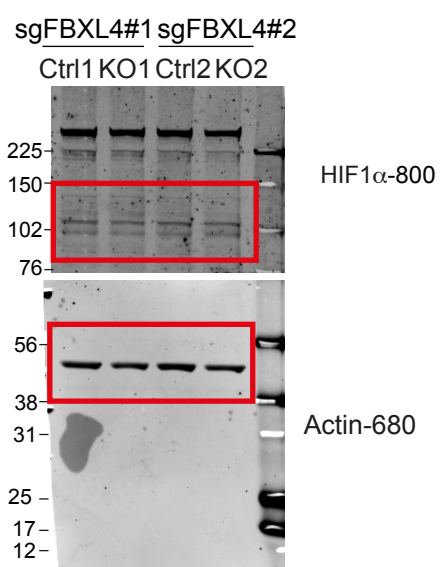

Supplement: Supplementary file 7 — Source Data for Figure 3 [file EMBJ-42-e112799-s009.zip › EMBOJ-2022-112799R_Figure3/Figure 3H/Figure 3H-Western Blot.pdf]

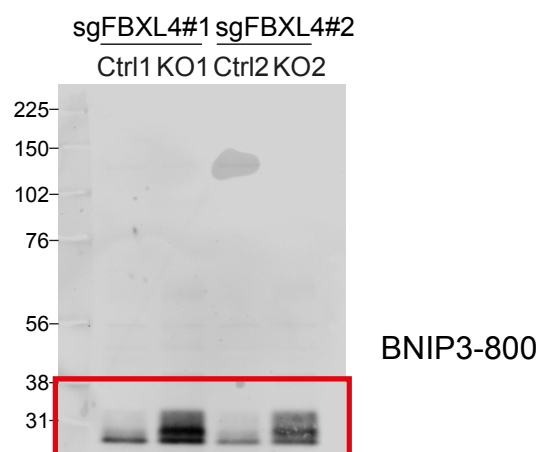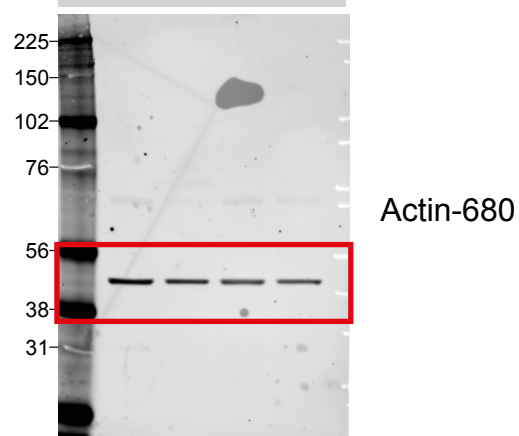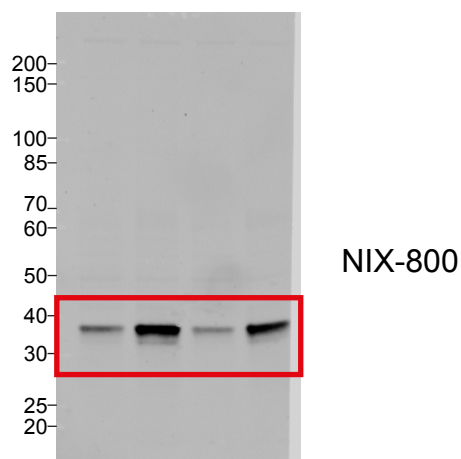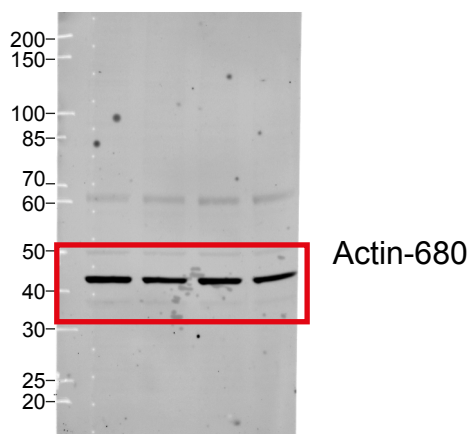

Supplement: Supplementary file 7 — Source Data for Figure 3 [file EMBJ-42-e112799-s009.zip › EMBOJ-2022-112799R_Figure3/Figure 3A/Figure 3A Western blot.pdf]

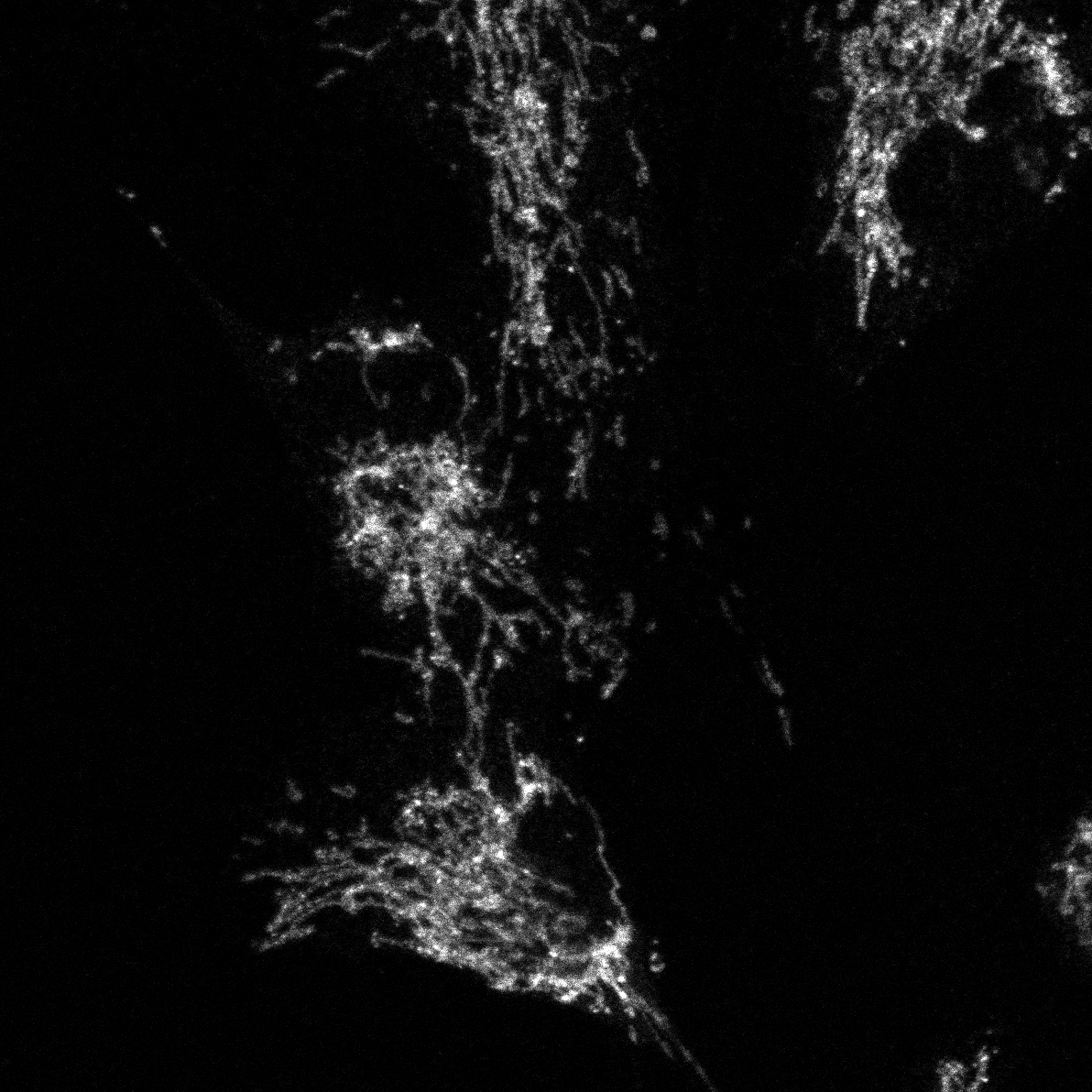

Supplement: Supplementary file 7 — Source Data for Figure 3 [file EMBJ-42-e112799-s009.zip › EMBOJ-2022-112799R_Figure3/Figure 3C/Nix/Control2-TOMM20.tif]

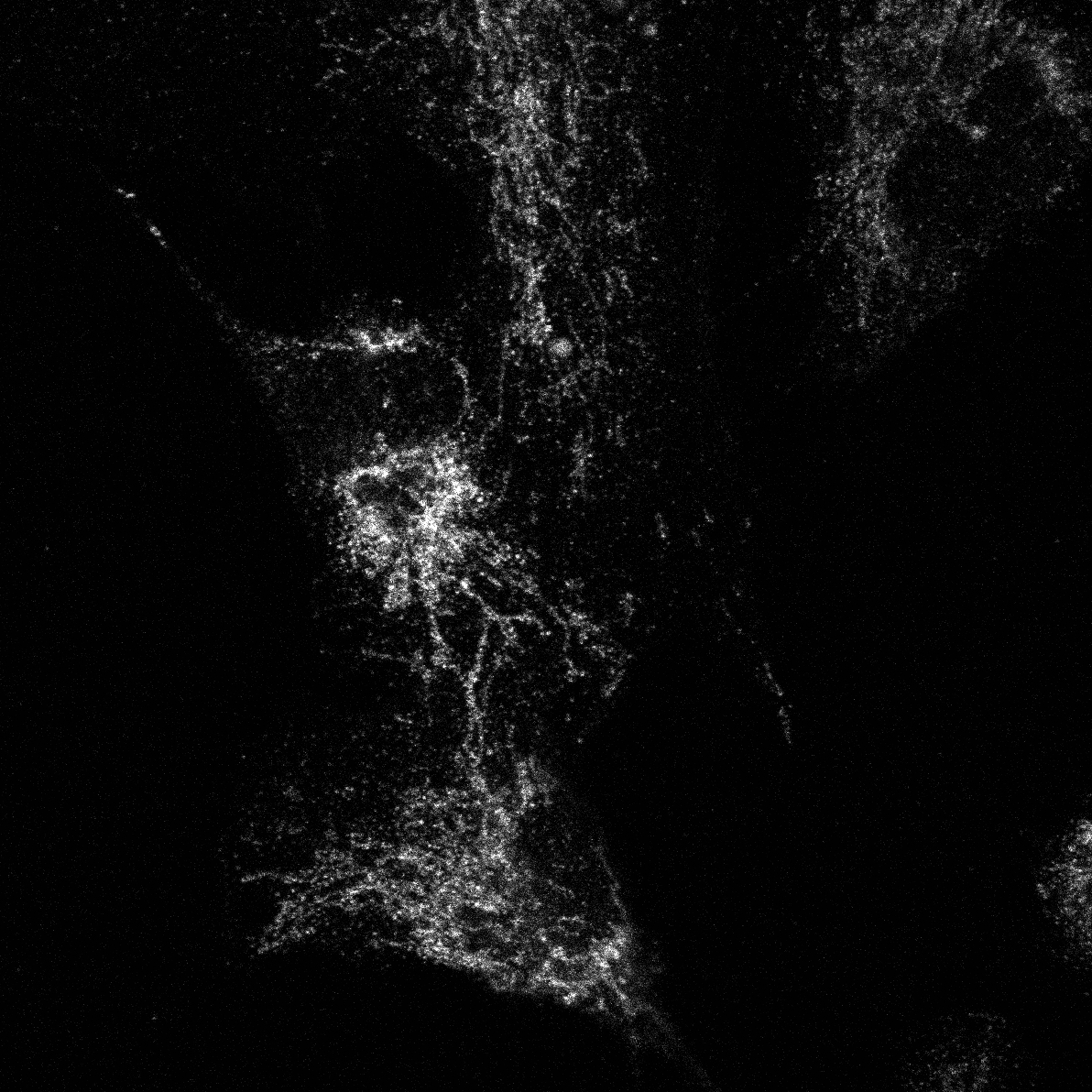

Supplement: Supplementary file 7 — Source Data for Figure 3 [file EMBJ-42-e112799-s009.zip › EMBOJ-2022-112799R_Figure3/Figure 3C/Nix/Control2- NIX.tif]

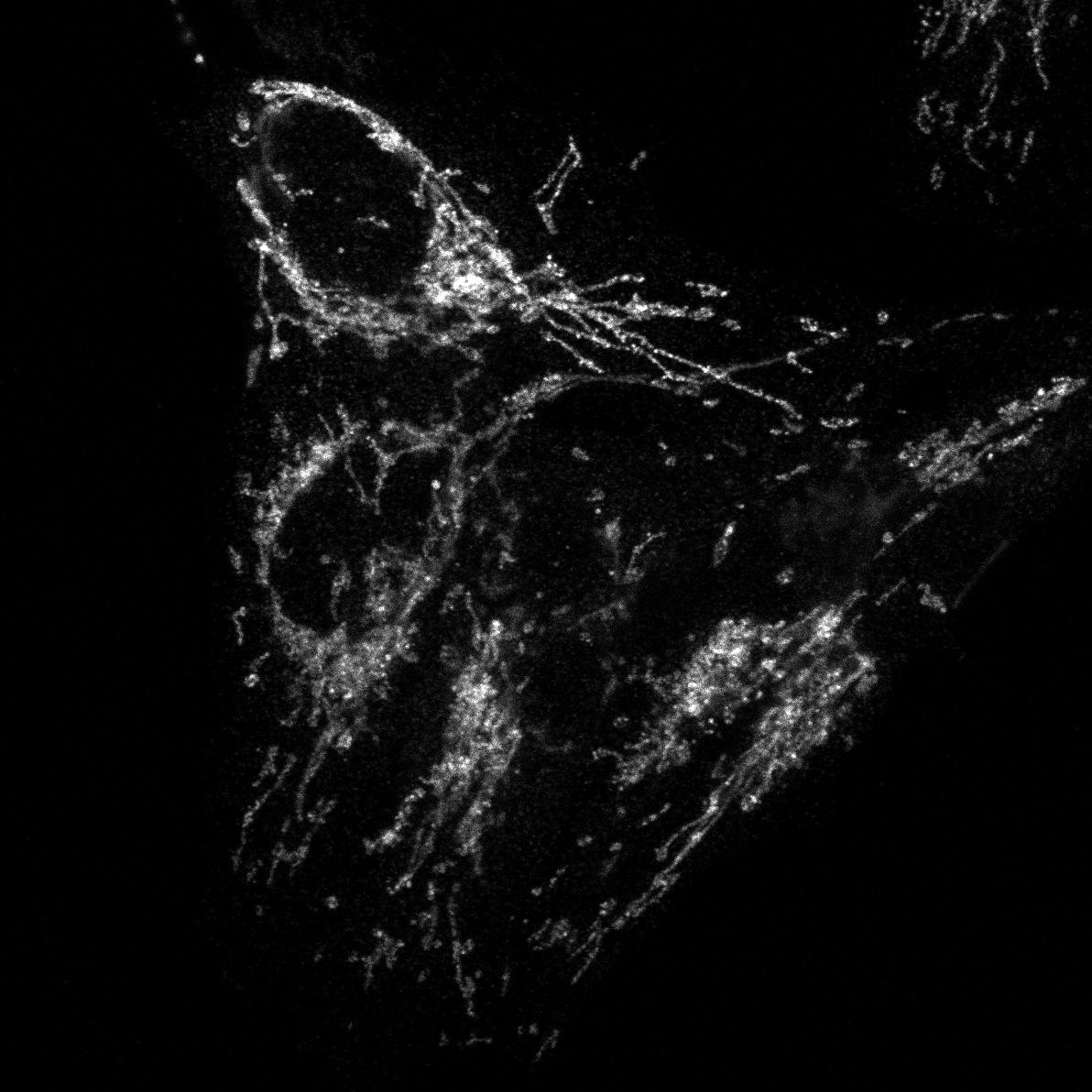

Supplement: Supplementary file 7 — Source Data for Figure 3 [file EMBJ-42-e112799-s009.zip › EMBOJ-2022-112799R_Figure3/Figure 3C/Nix/KO2-Nix.tif]

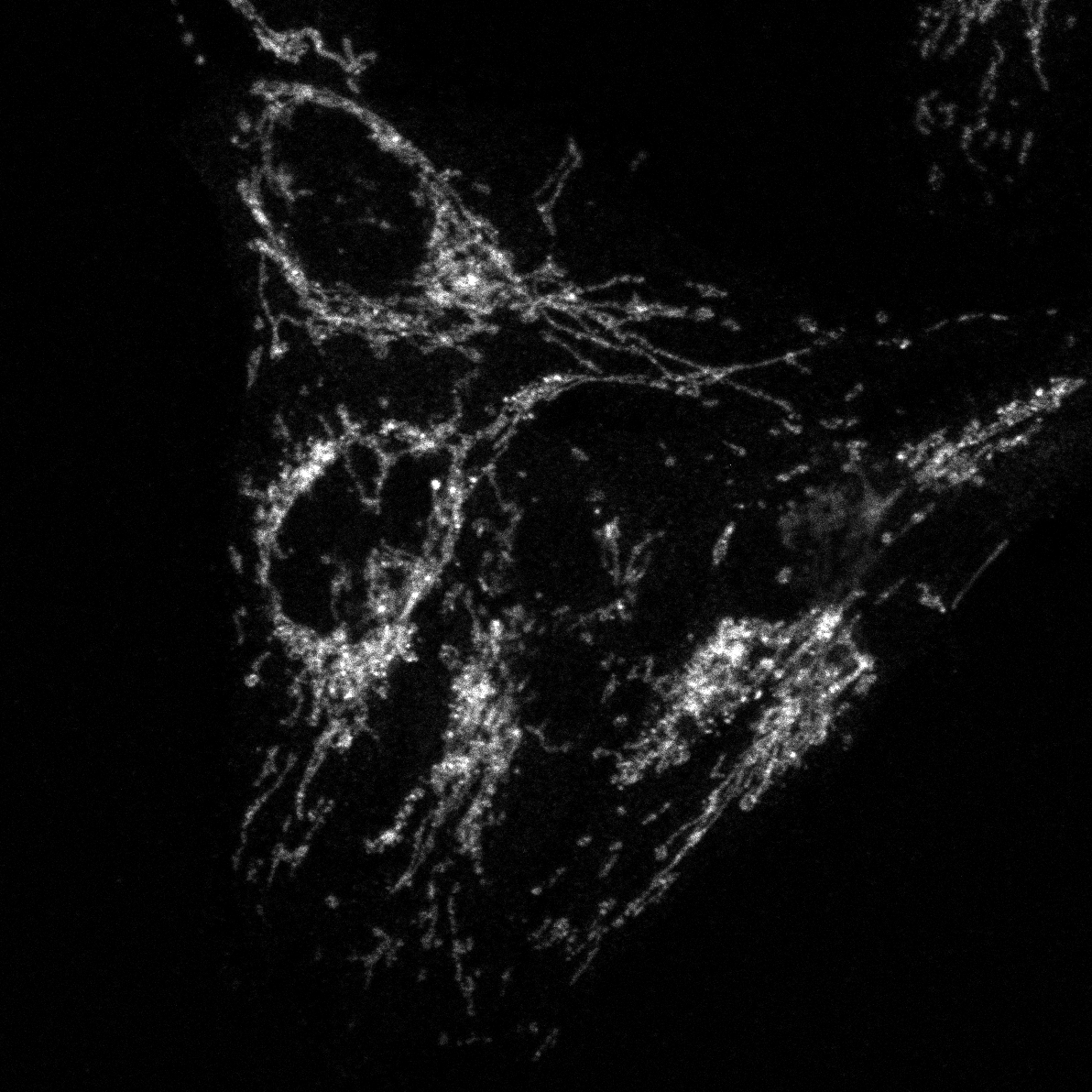

Supplement: Supplementary file 7 — Source Data for Figure 3 [file EMBJ-42-e112799-s009.zip › EMBOJ-2022-112799R_Figure3/Figure 3C/Nix/KO2-TOMM20.tif]

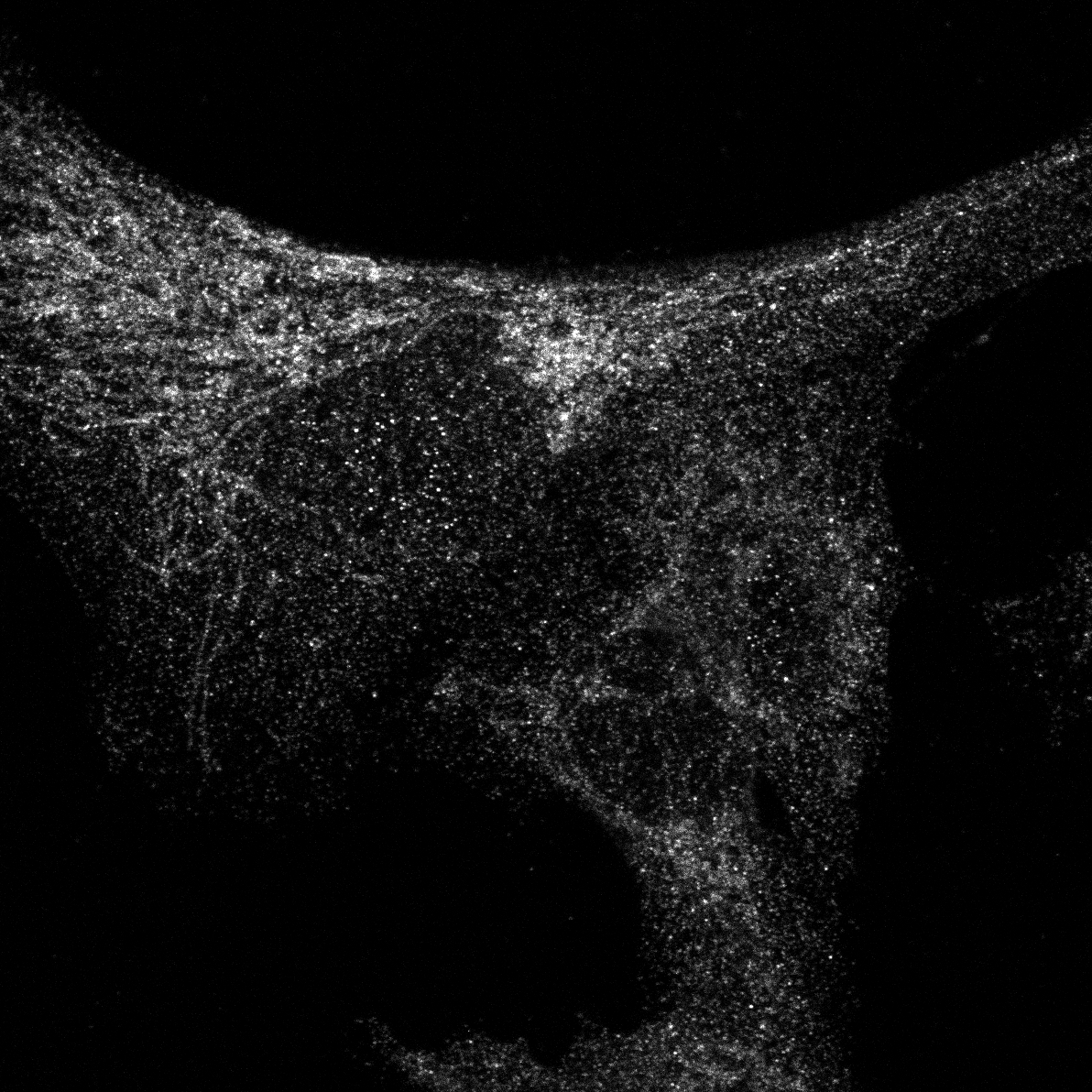

Supplement: Supplementary file 7 — Source Data for Figure 3 [file EMBJ-42-e112799-s009.zip › EMBOJ-2022-112799R_Figure3/Figure 3C/BNIP3/Control 2 BNIP3.tif]

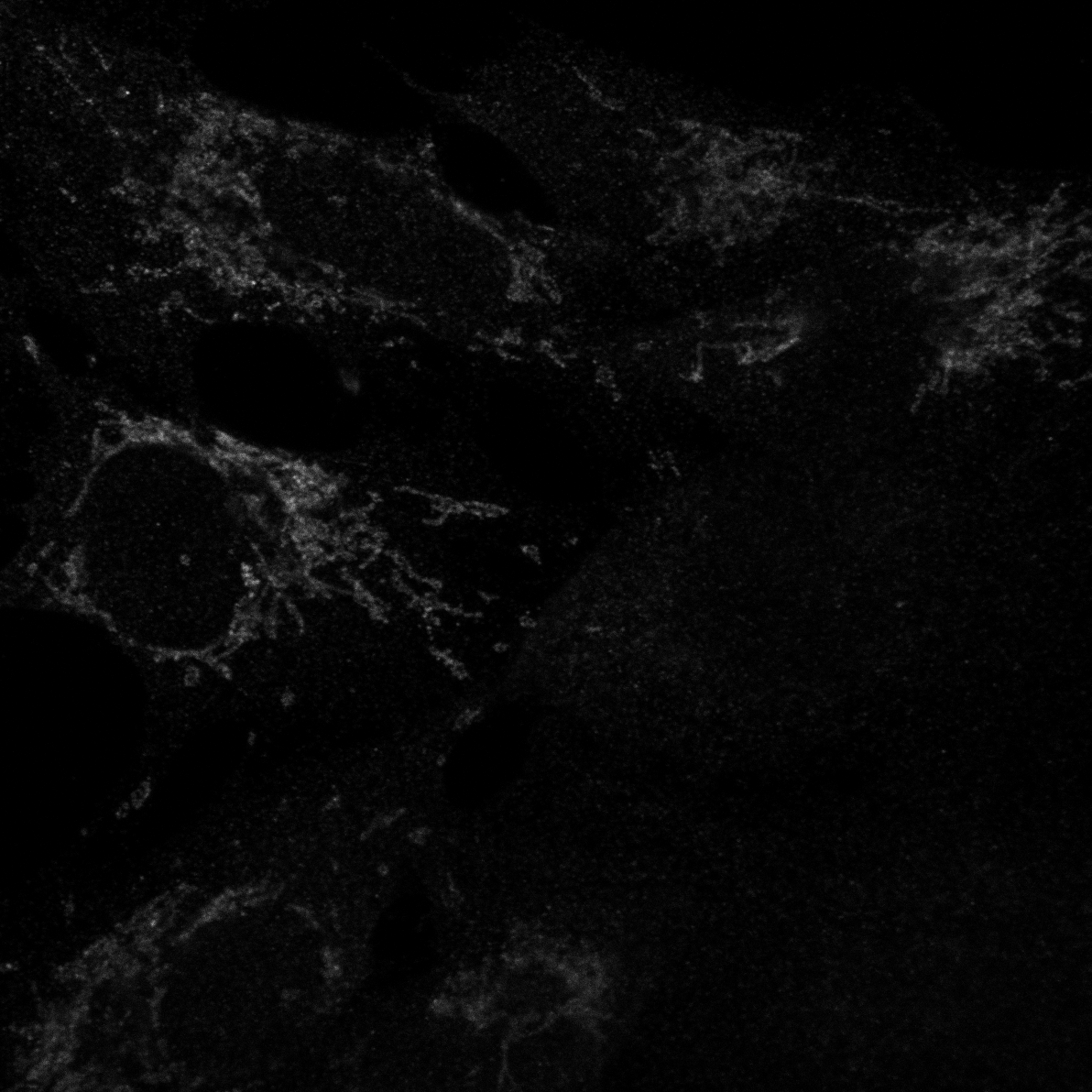

Supplement: Supplementary file 7 — Source Data for Figure 3 [file EMBJ-42-e112799-s009.zip › EMBOJ-2022-112799R_Figure3/Figure 3C/BNIP3/KO2- BNIP3.tif]

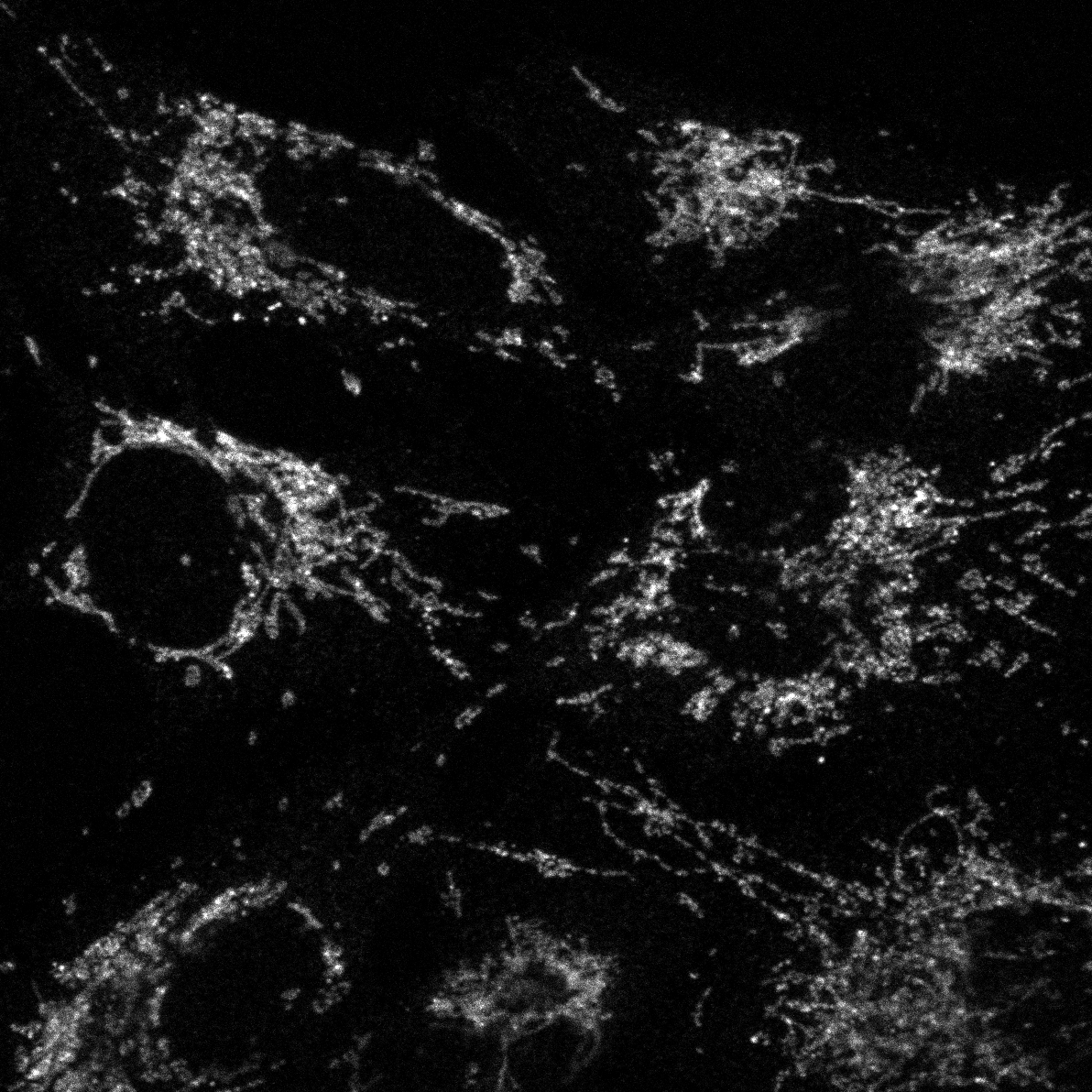

Supplement: Supplementary file 7 — Source Data for Figure 3 [file EMBJ-42-e112799-s009.zip › EMBOJ-2022-112799R_Figure3/Figure 3C/BNIP3/KO2- TOMM20.tif]

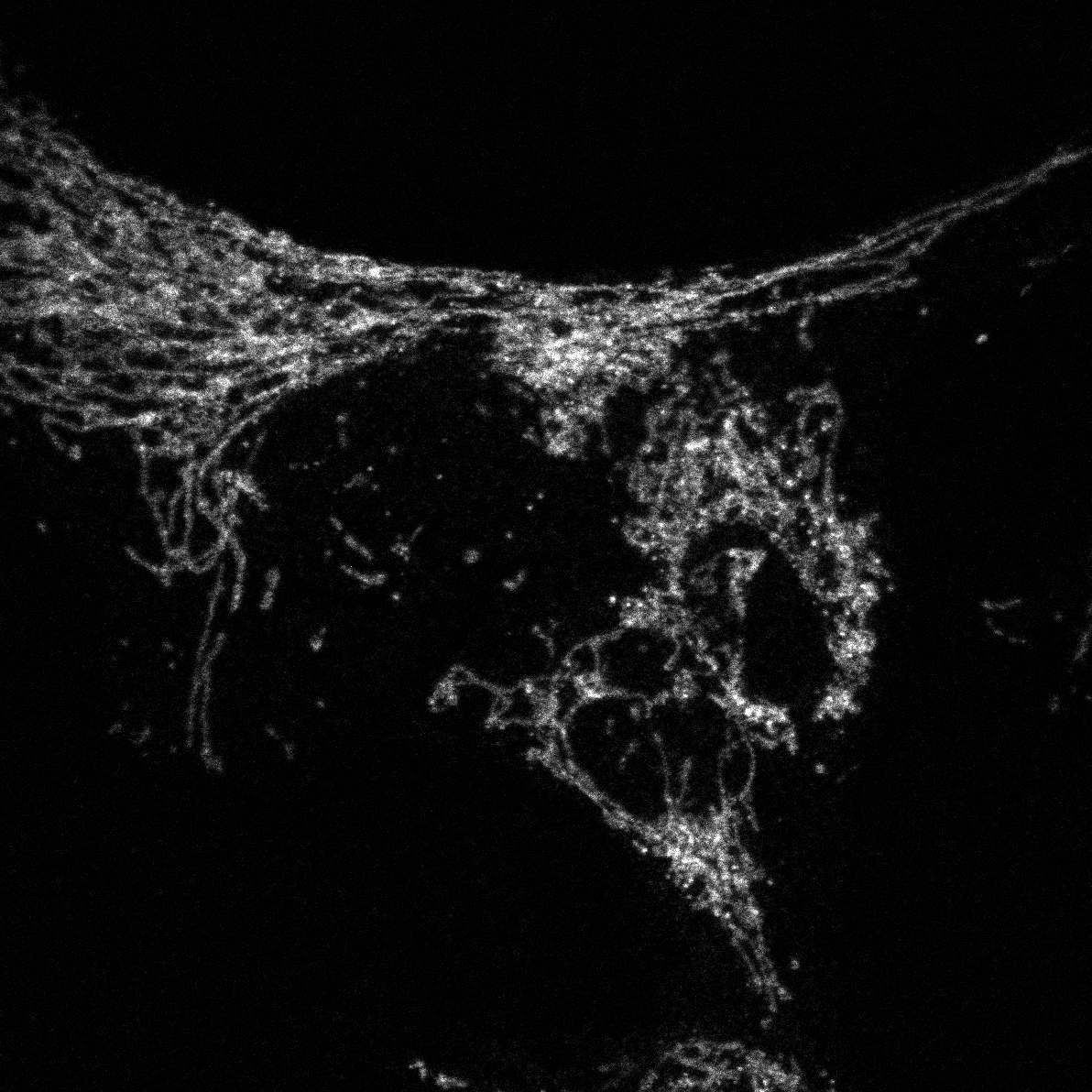

Supplement: Supplementary file 7 — Source Data for Figure 3 [file EMBJ-42-e112799-s009.zip › EMBOJ-2022-112799R_Figure3/Figure 3C/BNIP3/Control 2 TOMM20.tif]

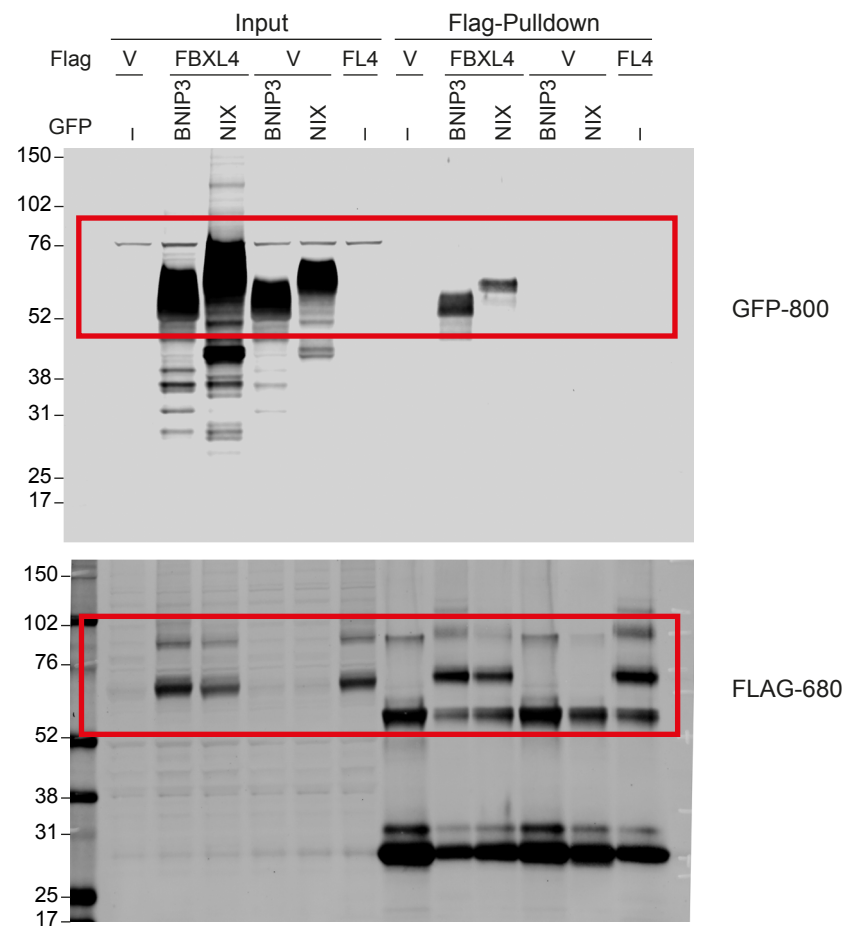

Supplement: Supplementary file 8 — Source Data for Figure 4 [file EMBJ-42-e112799-s003.zip › EMBOJ-2022-112799R_Figure4/Figure 4B/Figure 4B-CoIP-western blot.pdf]

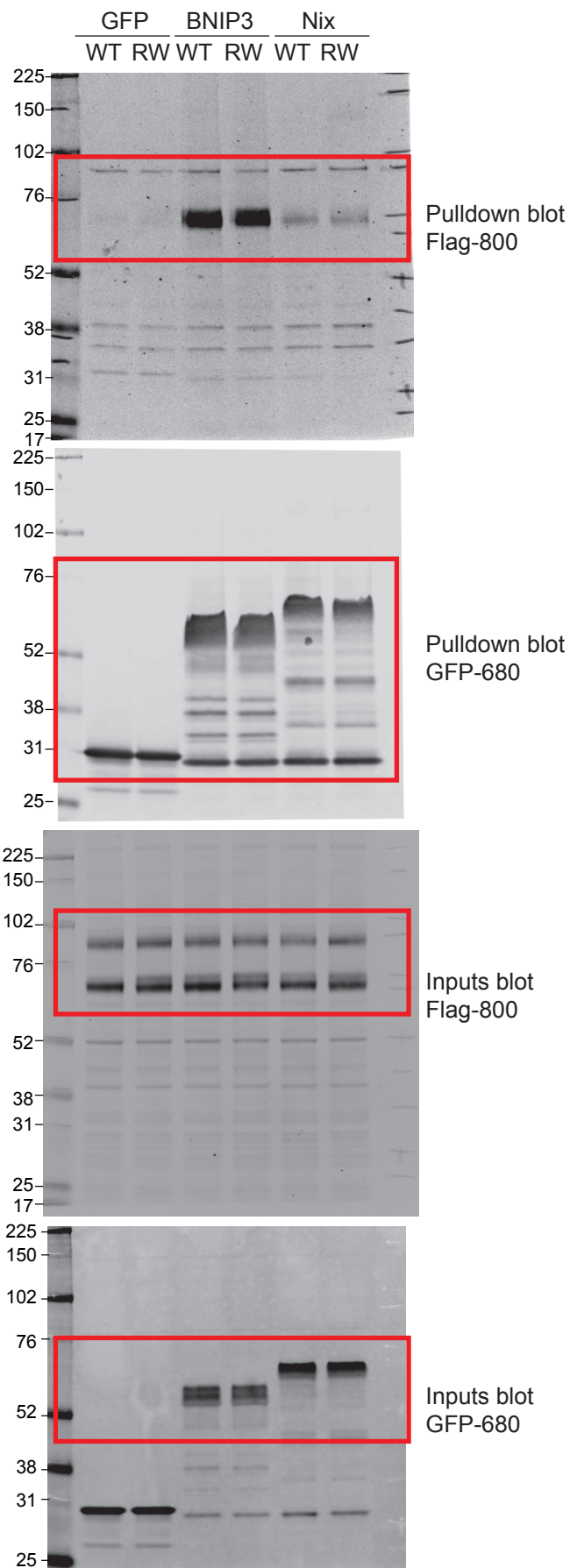

Supplement: Supplementary file 8 — Source Data for Figure 4 [file EMBJ-42-e112799-s003.zip › EMBOJ-2022-112799R_Figure4/Figure 4C/Figure 4C-Western blot-COIP.pdf]

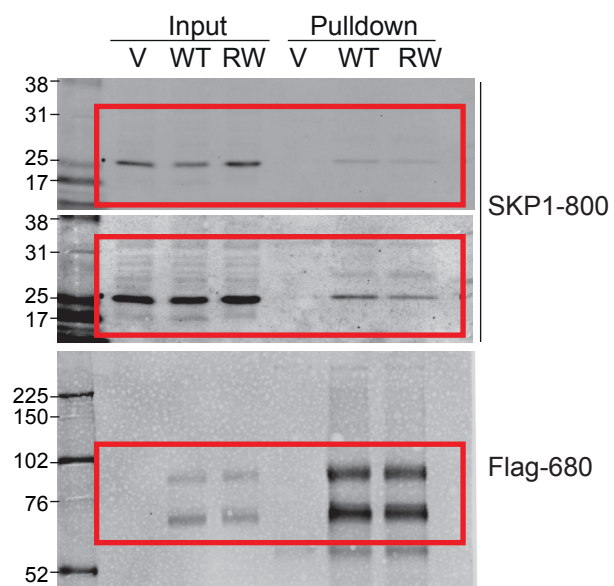

Supplement: Supplementary file 8 — Source Data for Figure 4 [file EMBJ-42-e112799-s003.zip › EMBOJ-2022-112799R_Figure4/Figure 4D/Figure 4D-Western Blot-IP.pdf]

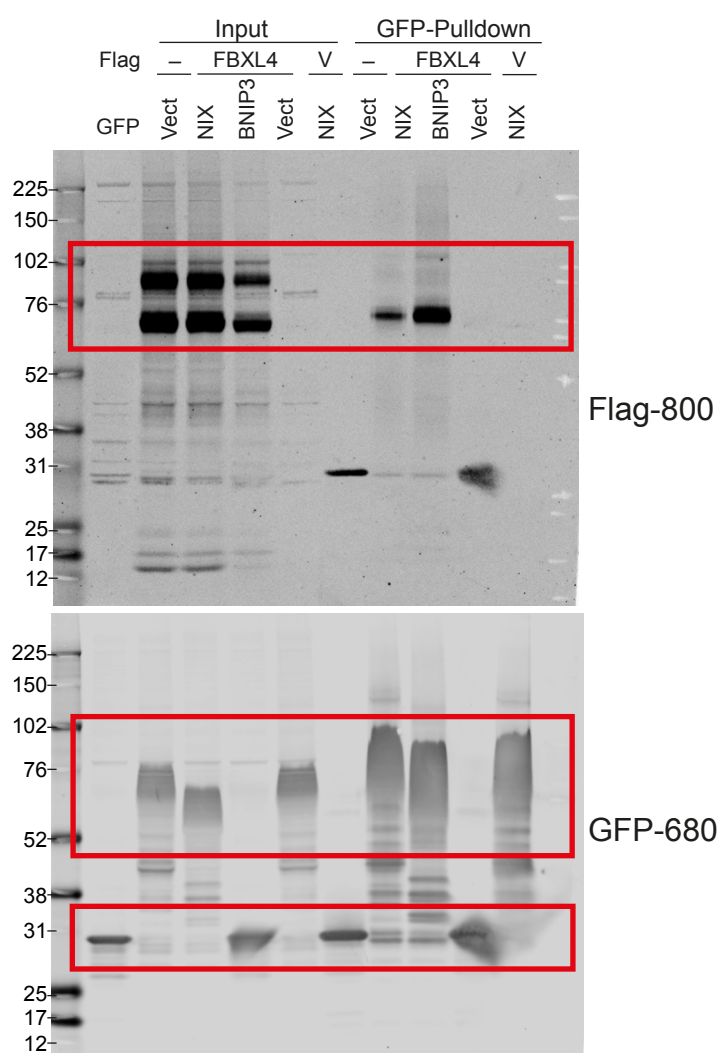

Supplement: Supplementary file 8 — Source Data for Figure 4 [file EMBJ-42-e112799-s003.zip › EMBOJ-2022-112799R_Figure4/Figure 4A/Figure 4A Co-IP western blot.pdf]

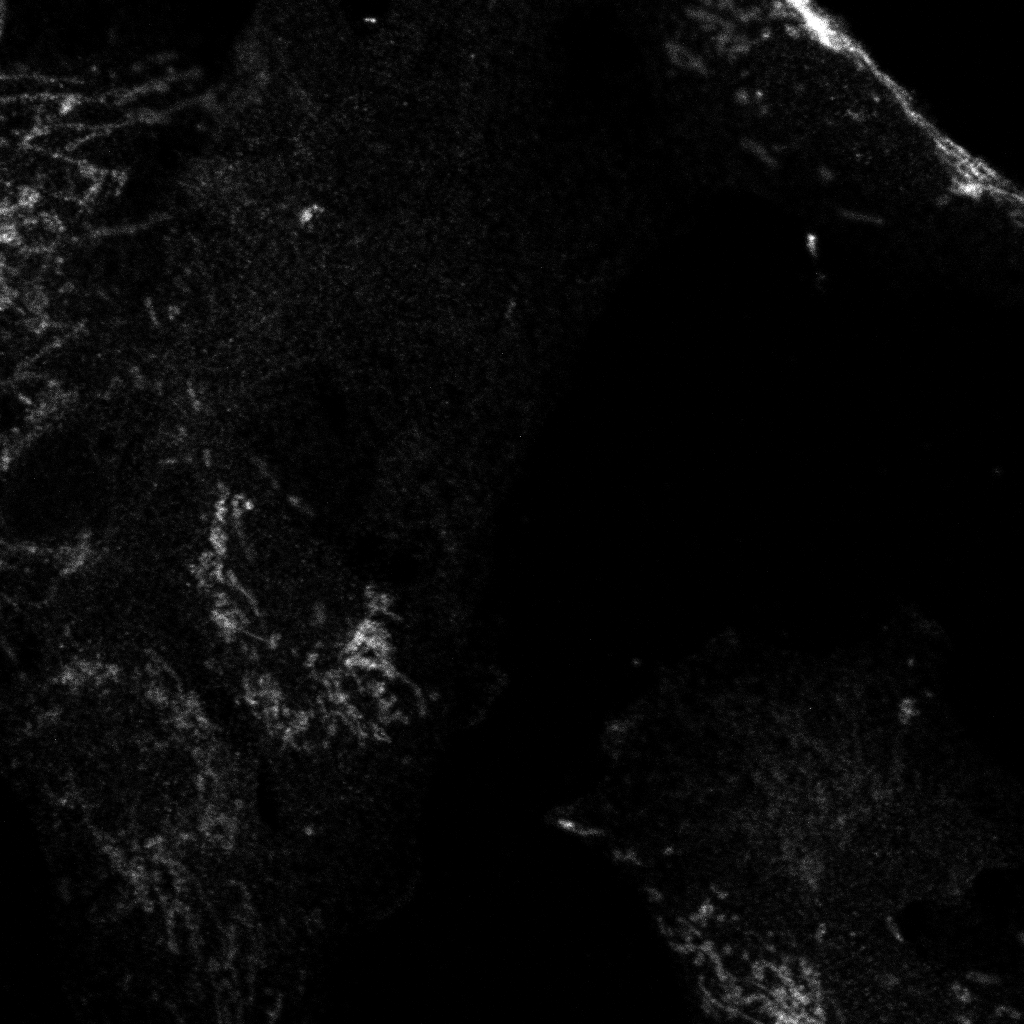

Supplement: Supplementary file 8 — Source Data for Figure 4 [file EMBJ-42-e112799-s003.zip › EMBOJ-2022-112799R_Figure4/Figure 4F/KO2_WT-BNIP3.tif]

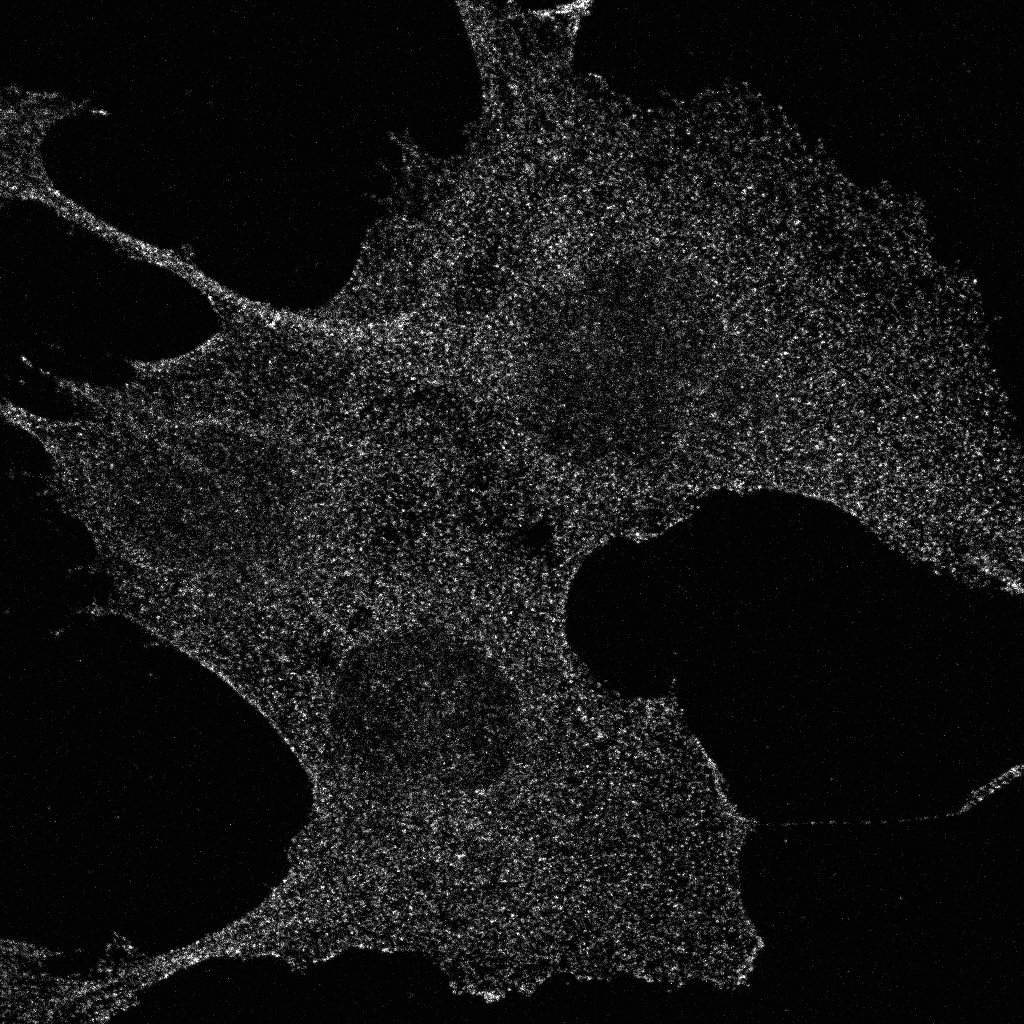

Supplement: Supplementary file 8 — Source Data for Figure 4 [file EMBJ-42-e112799-s003.zip › EMBOJ-2022-112799R_Figure4/Figure 4F/KO2_UT-FLAG.tif]

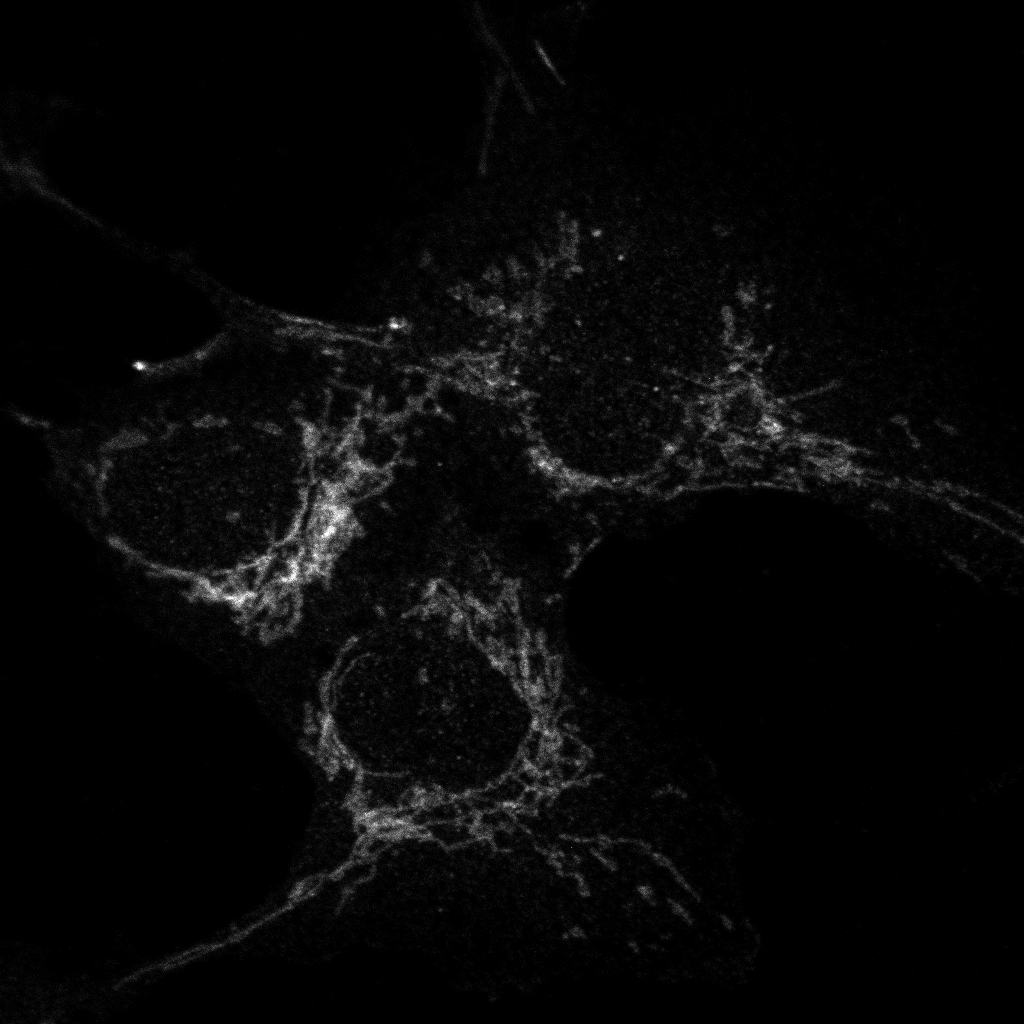

Supplement: Supplementary file 8 — Source Data for Figure 4 [file EMBJ-42-e112799-s003.zip › EMBOJ-2022-112799R_Figure4/Figure 4F/KO2_UT-BNIP3.tif]

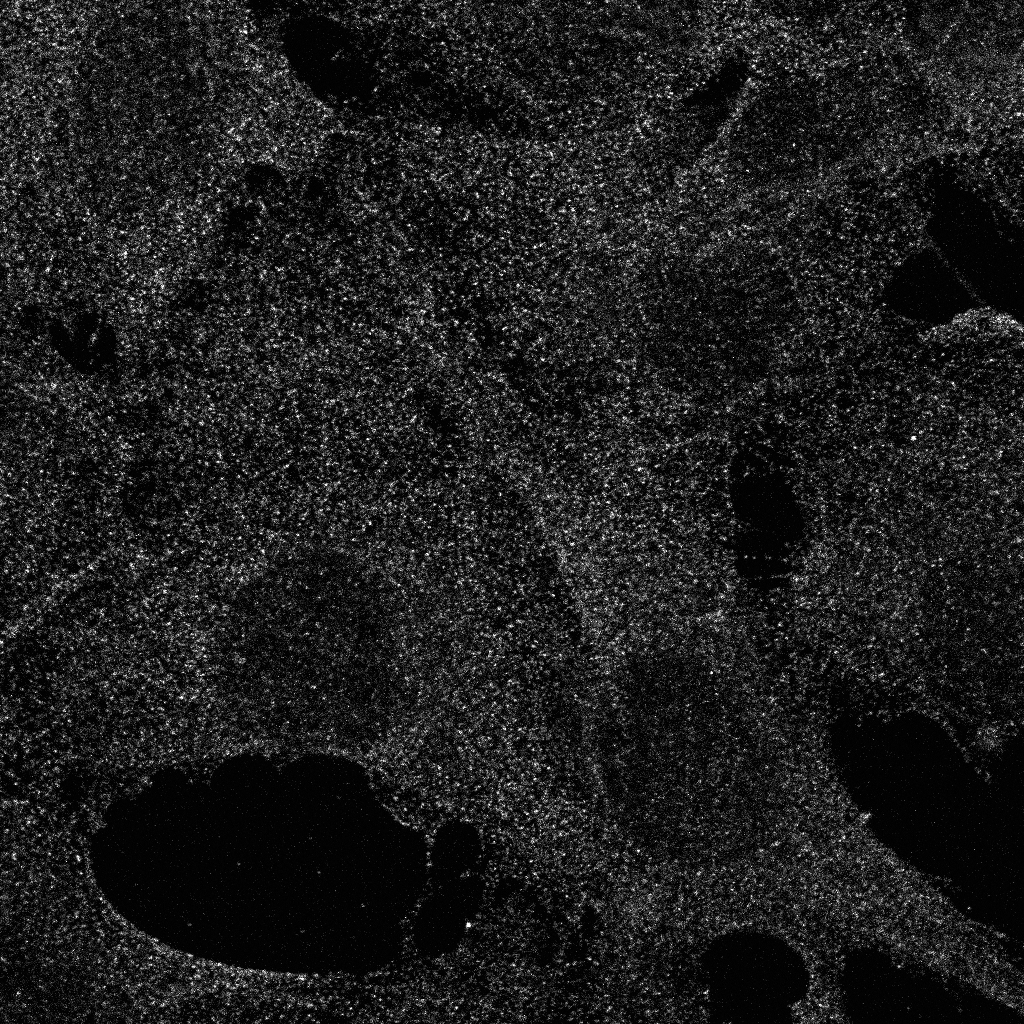

Supplement: Supplementary file 8 — Source Data for Figure 4 [file EMBJ-42-e112799-s003.zip › EMBOJ-2022-112799R_Figure4/Figure 4F/Ctrl2_UT-FLAG.tif]

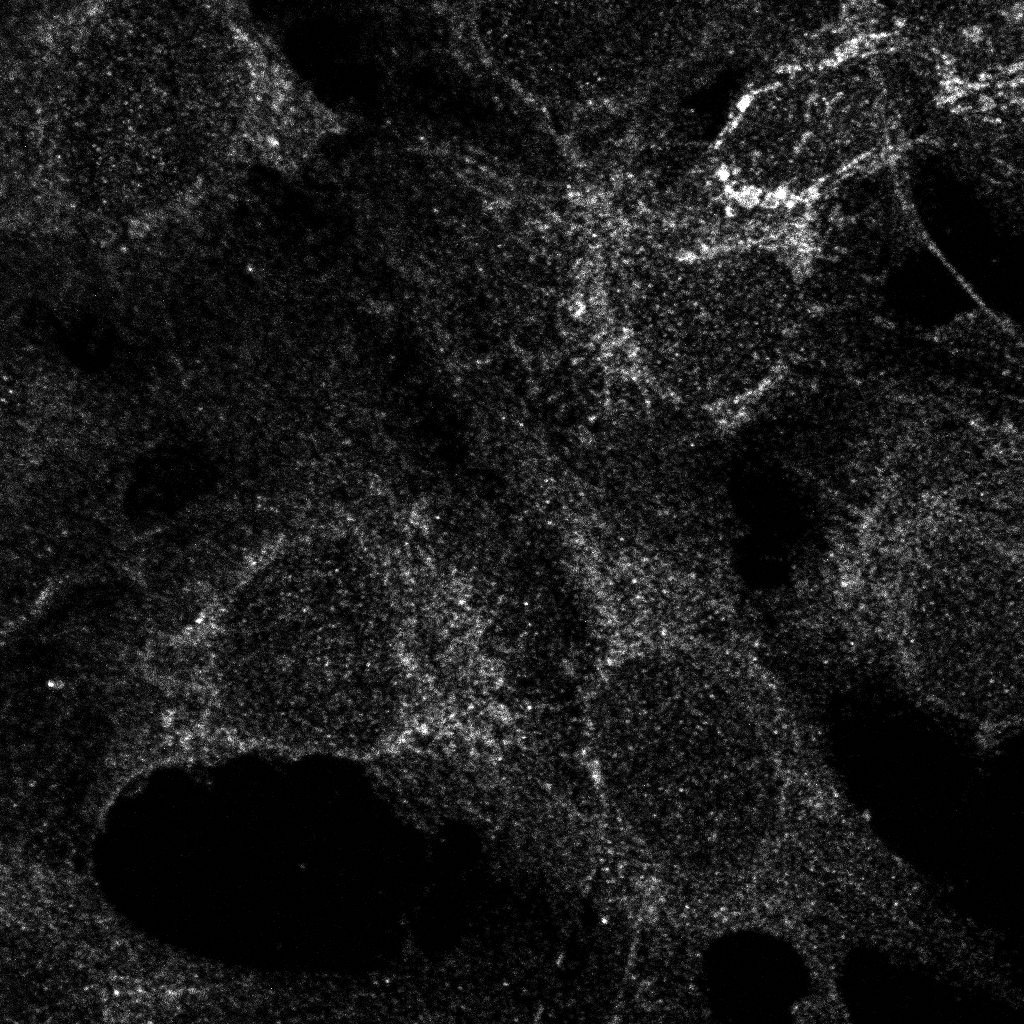

Supplement: Supplementary file 8 — Source Data for Figure 4 [file EMBJ-42-e112799-s003.zip › EMBOJ-2022-112799R_Figure4/Figure 4F/Ctrl 2_UT-BNIP3.tif]

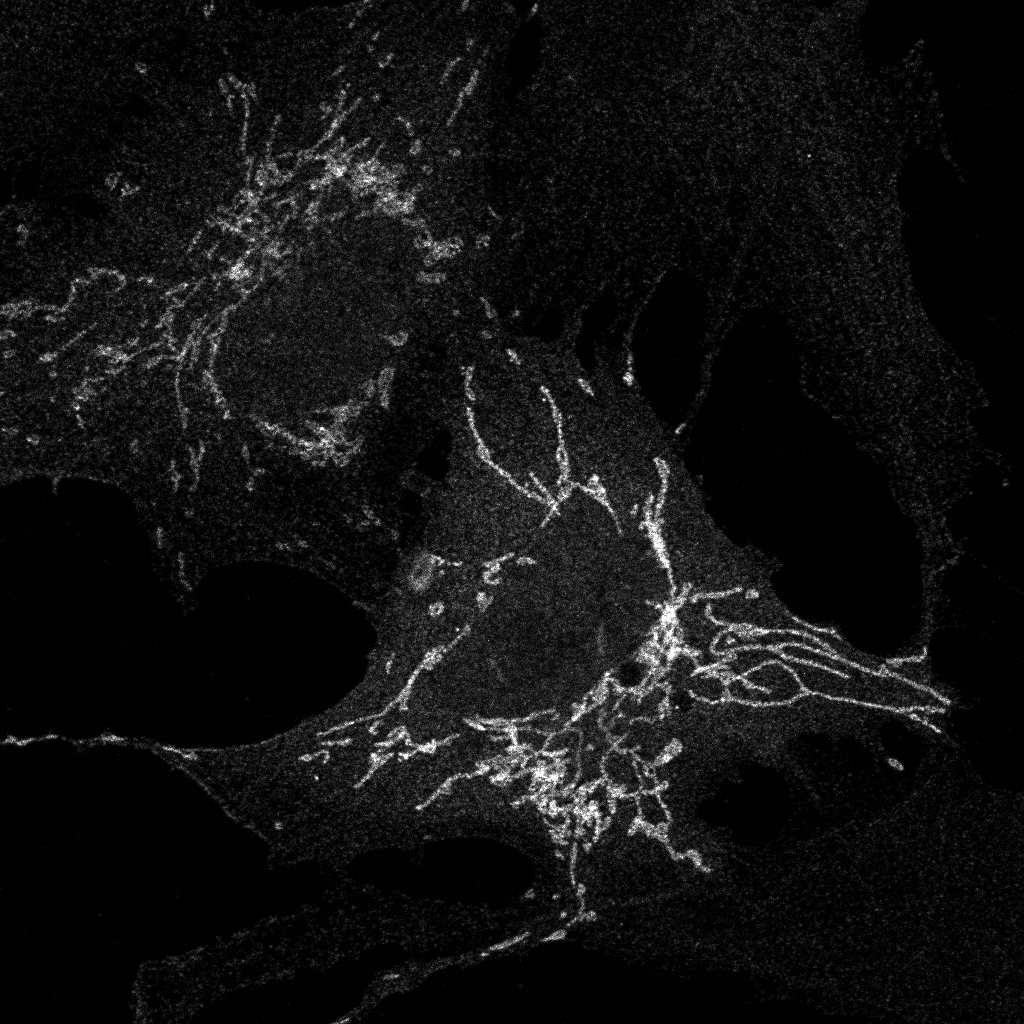

Supplement: Supplementary file 8 — Source Data for Figure 4 [file EMBJ-42-e112799-s003.zip › EMBOJ-2022-112799R_Figure4/Figure 4F/KO2_RW-FLAG.tif]

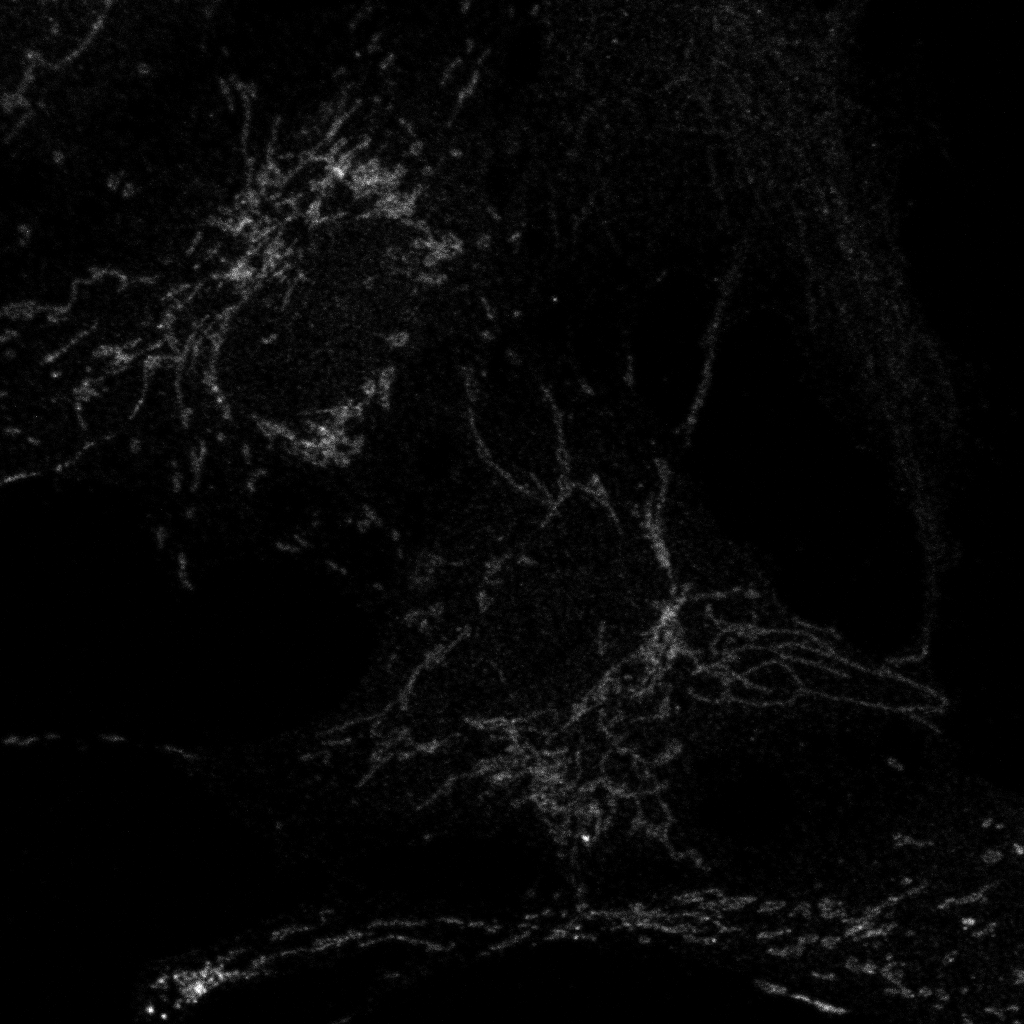

Supplement: Supplementary file 8 — Source Data for Figure 4 [file EMBJ-42-e112799-s003.zip › EMBOJ-2022-112799R_Figure4/Figure 4F/KO2_RW-BNIP3.tif]

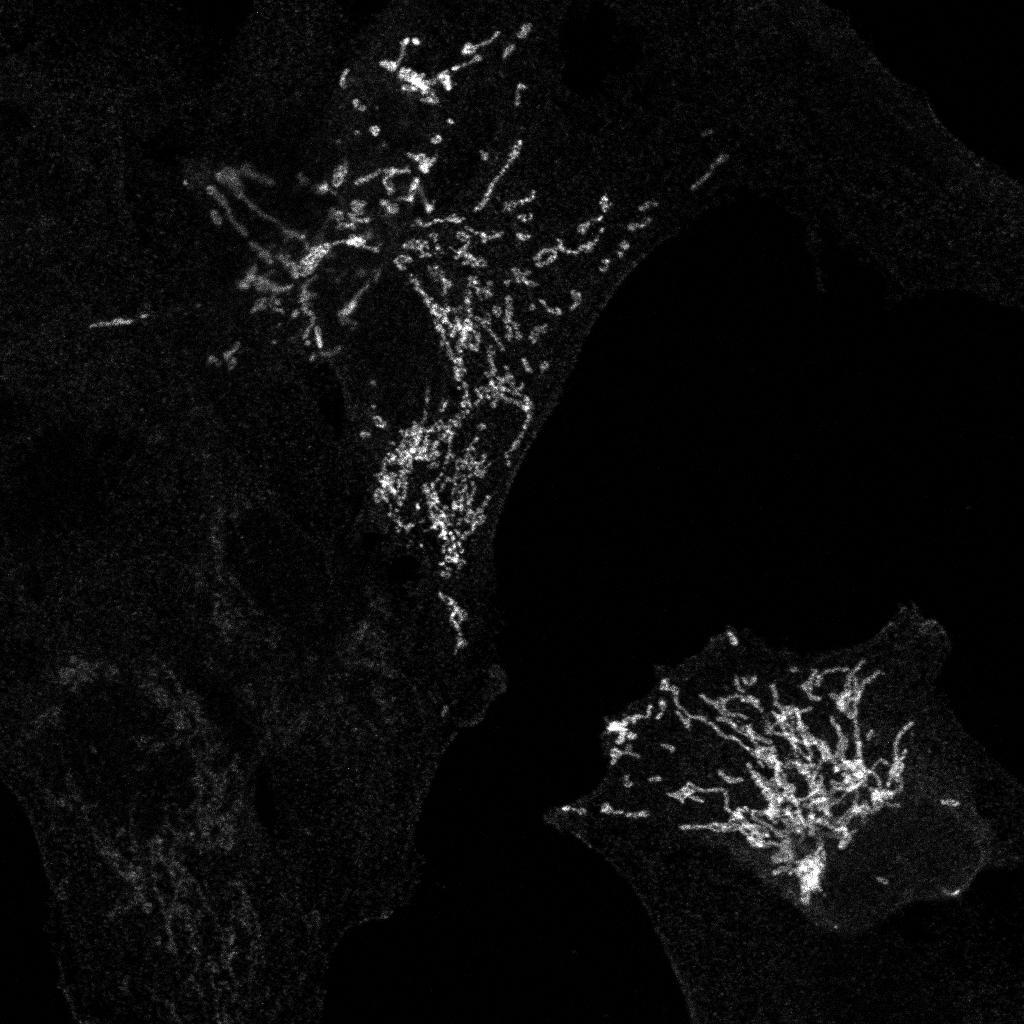

Supplement: Supplementary file 8 — Source Data for Figure 4 [file EMBJ-42-e112799-s003.zip › EMBOJ-2022-112799R_Figure4/Figure 4F/KO2_WT-FLAG.tif]

Ctrl2 FBXL4 KO2

Vec Vec WT RW

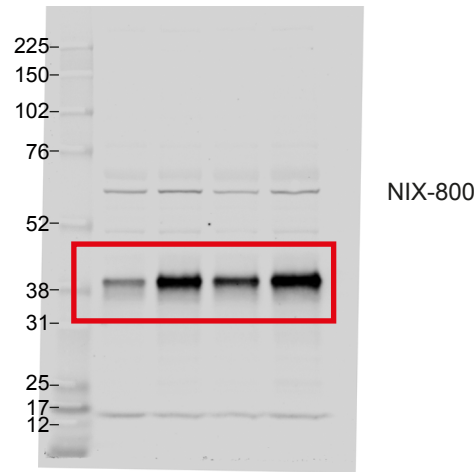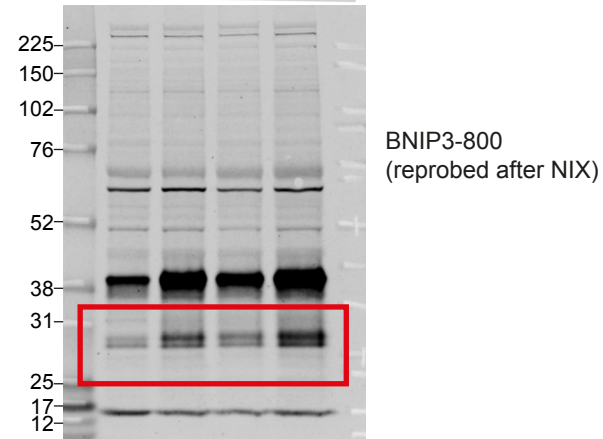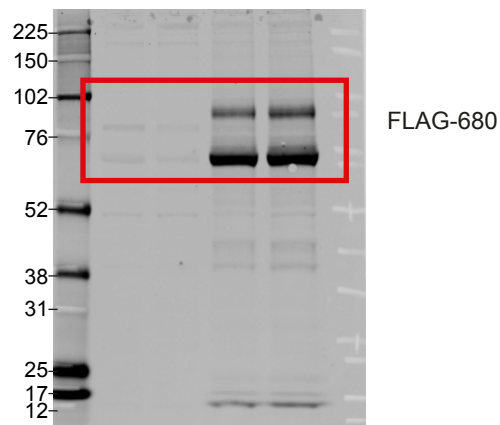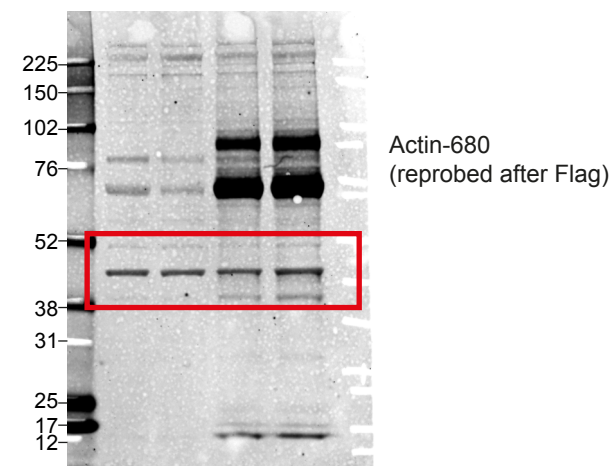

Supplement: Supplementary file 8 — Source Data for Figure 4 [file EMBJ-42-e112799-s003.zip › EMBOJ-2022-112799R_Figure4/Figure 4H/EMBOJ-2022-112799R-Figure_4H_Source_Data-sd.pdf]

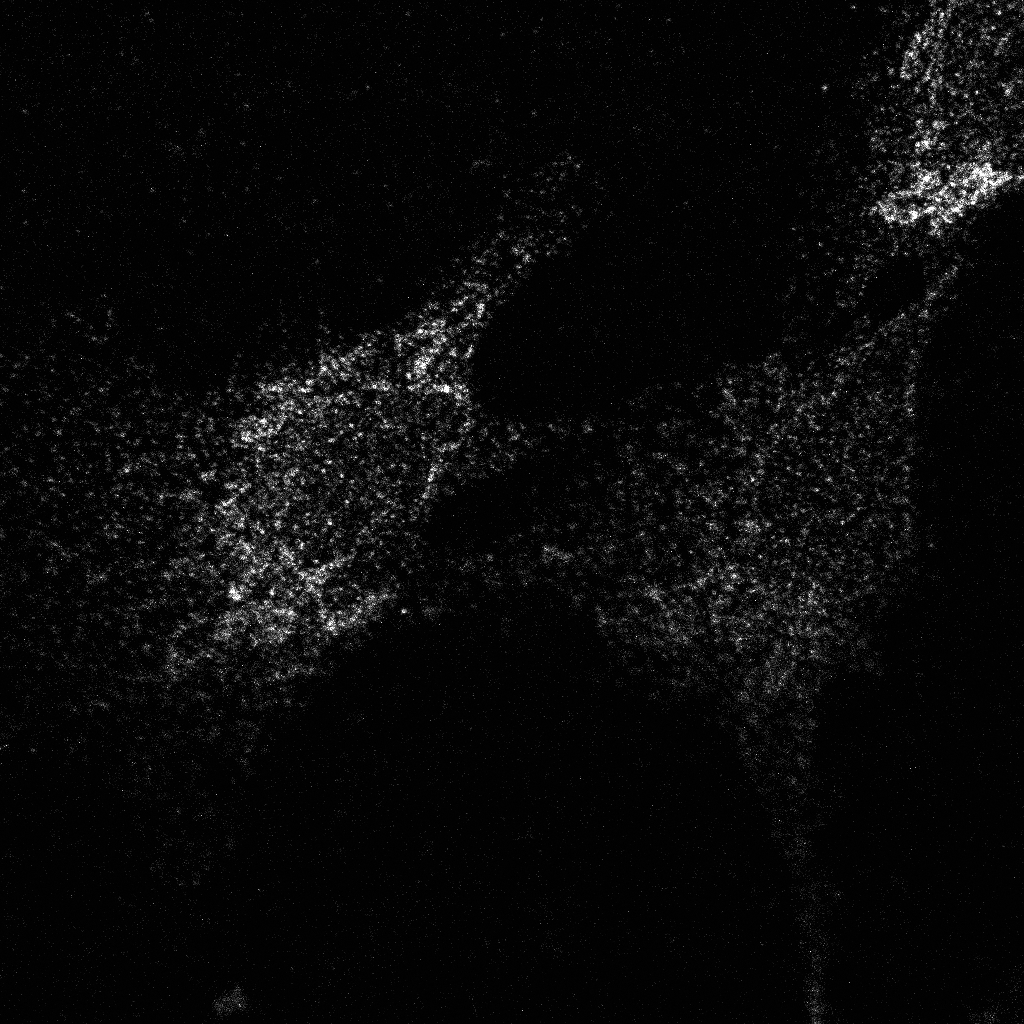

Supplement: Supplementary file 8 — Source Data for Figure 4 [file EMBJ-42-e112799-s003.zip › EMBOJ-2022-112799R_Figure4/Figure 4G/KO2_WT-Nix.tif]

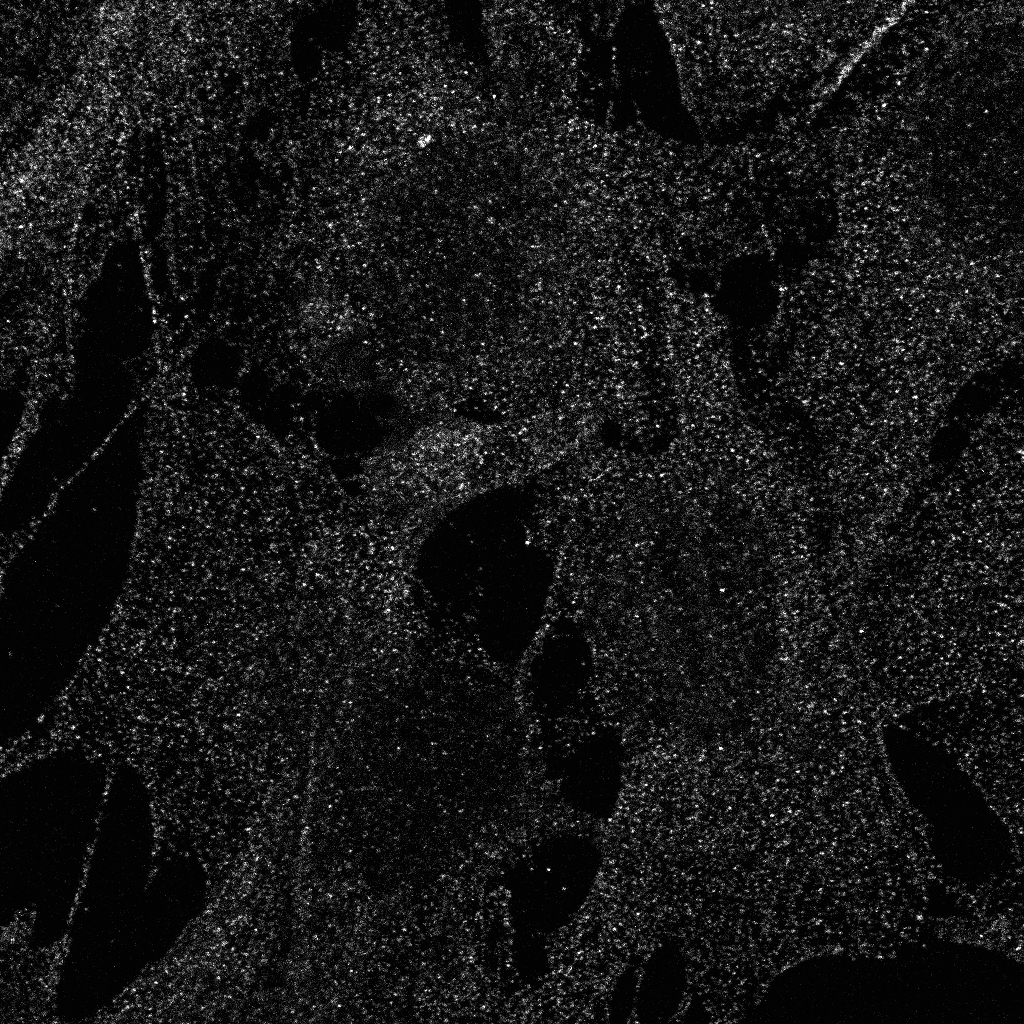

Supplement: Supplementary file 8 — Source Data for Figure 4 [file EMBJ-42-e112799-s003.zip › EMBOJ-2022-112799R_Figure4/Figure 4G/KO2_UT-FLAG.tif]

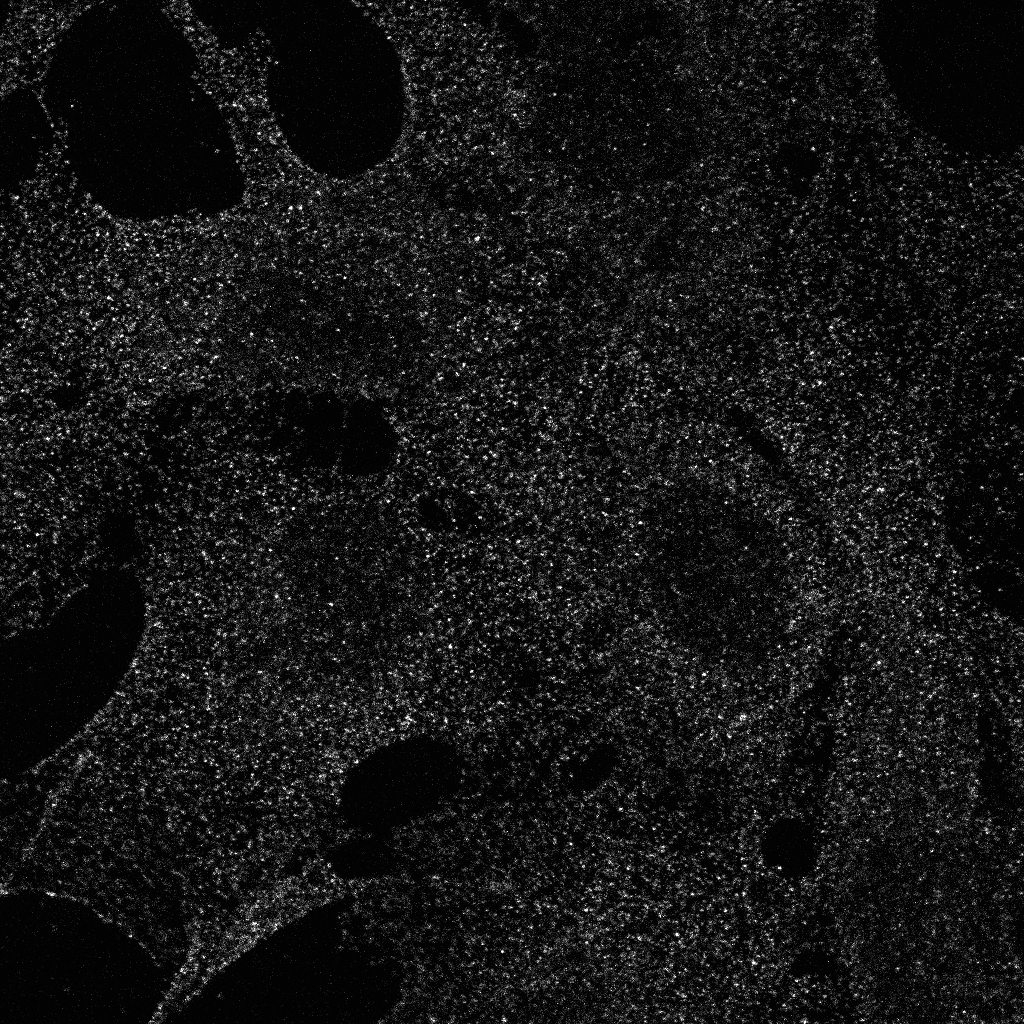

Supplement: Supplementary file 8 — Source Data for Figure 4 [file EMBJ-42-e112799-s003.zip › EMBOJ-2022-112799R_Figure4/Figure 4G/Ctrl2_UT-FLAG.tif]

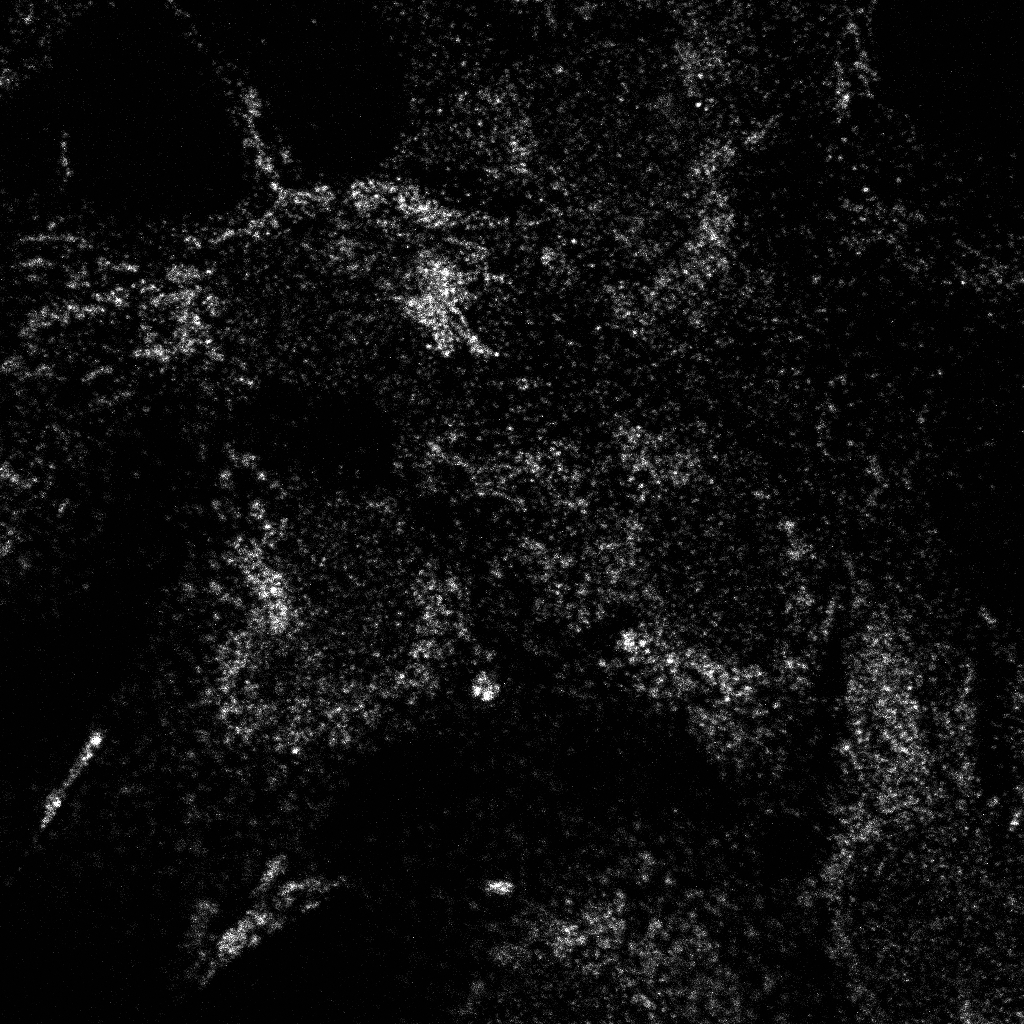

Supplement: Supplementary file 8 — Source Data for Figure 4 [file EMBJ-42-e112799-s003.zip › EMBOJ-2022-112799R_Figure4/Figure 4G/Ctrl2_UT-Nix.tif]

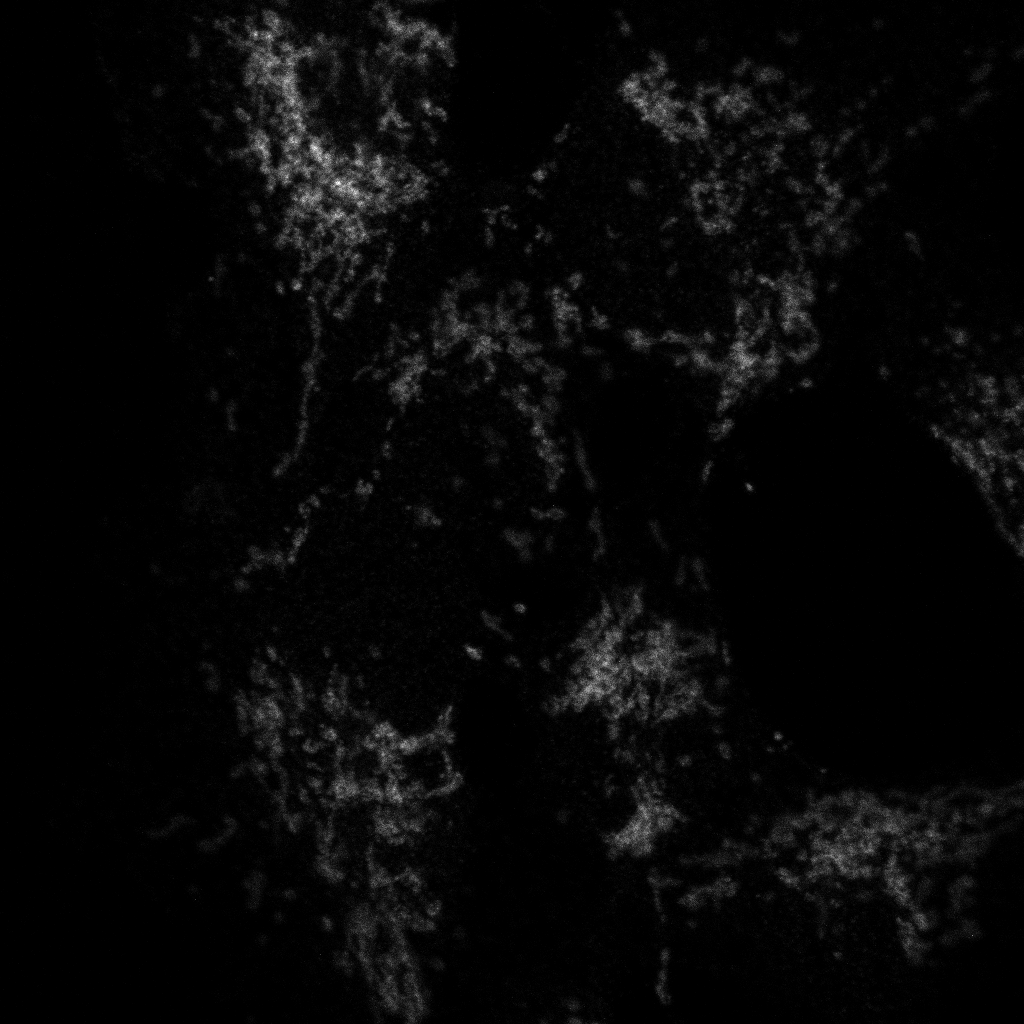

Supplement: Supplementary file 8 — Source Data for Figure 4 [file EMBJ-42-e112799-s003.zip › EMBOJ-2022-112799R_Figure4/Figure 4G/KO2_RW-Nix.tif]

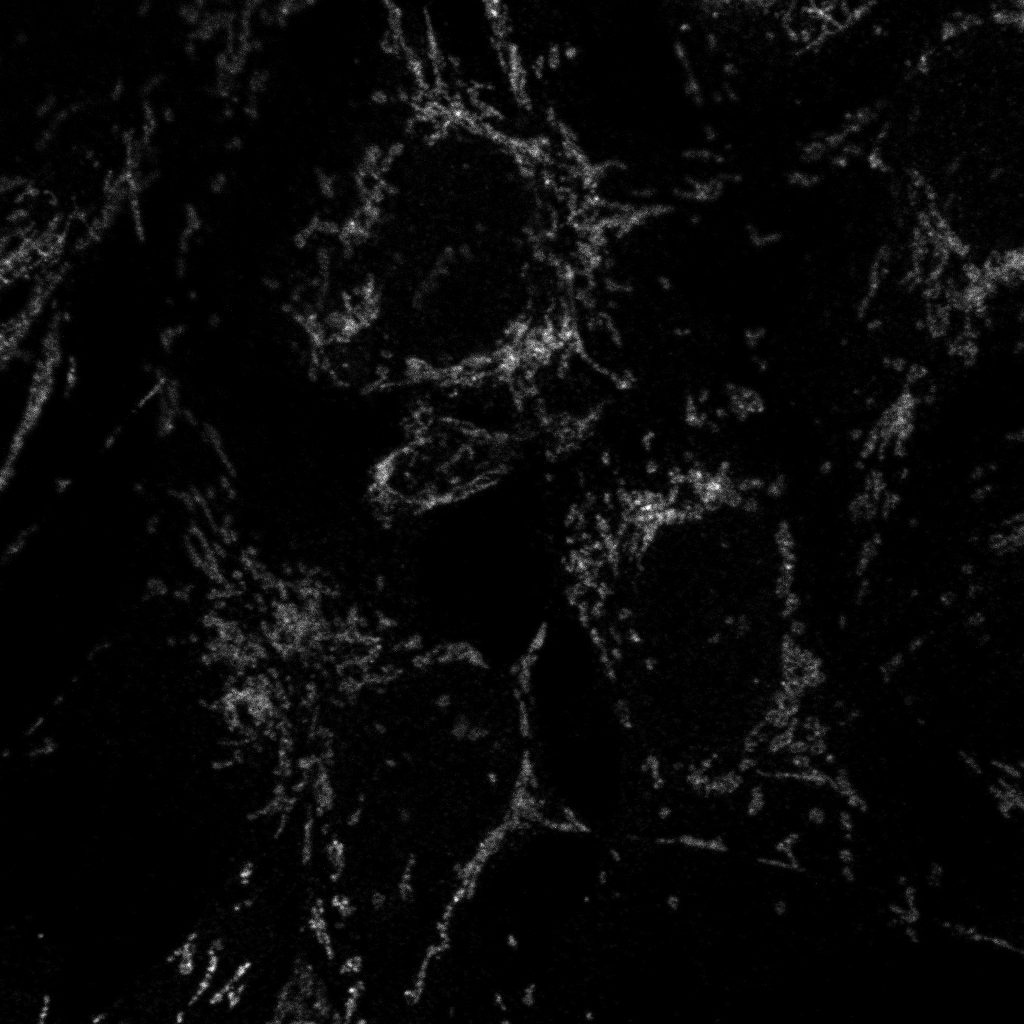

Supplement: Supplementary file 8 — Source Data for Figure 4 [file EMBJ-42-e112799-s003.zip › EMBOJ-2022-112799R_Figure4/Figure 4G/KO2_UT-NIX.tif]

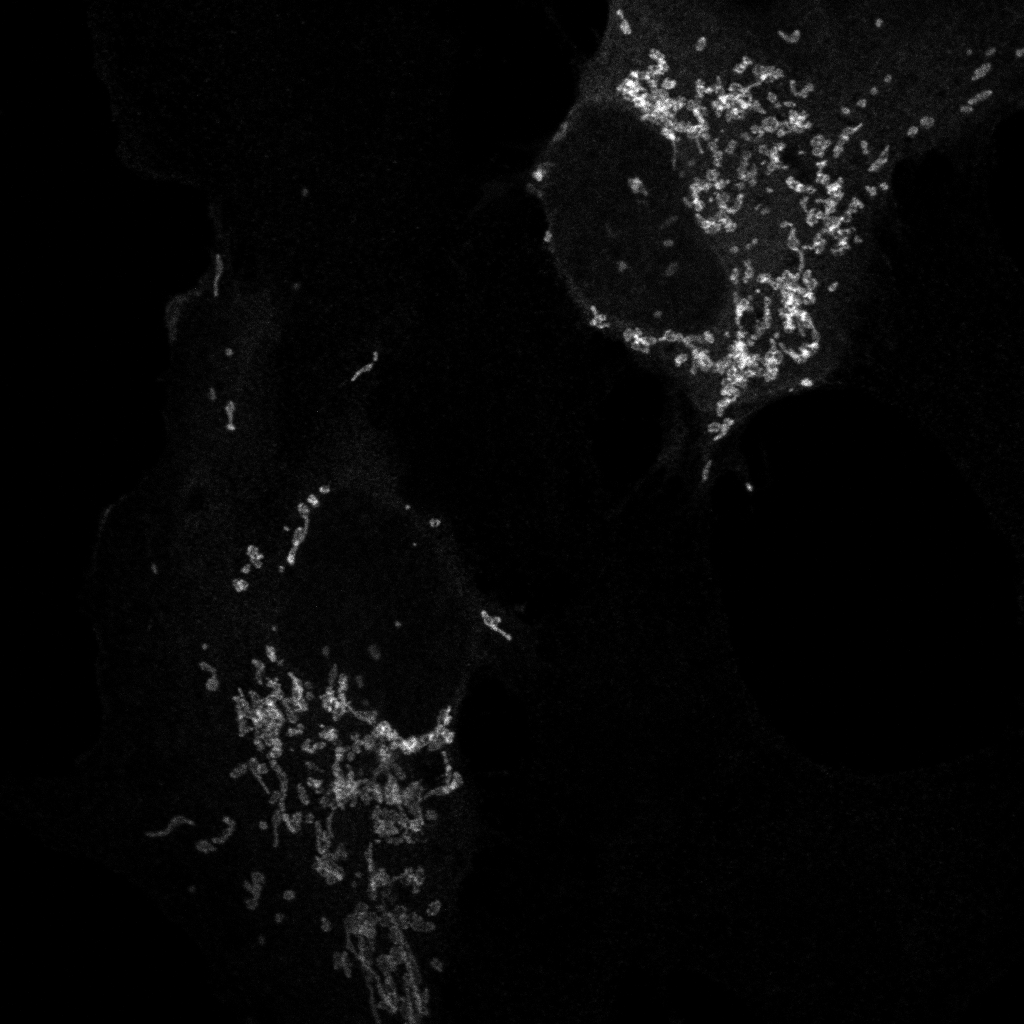

Supplement: Supplementary file 8 — Source Data for Figure 4 [file EMBJ-42-e112799-s003.zip › EMBOJ-2022-112799R_Figure4/Figure 4G/KO2_RW-FLAG.tif]

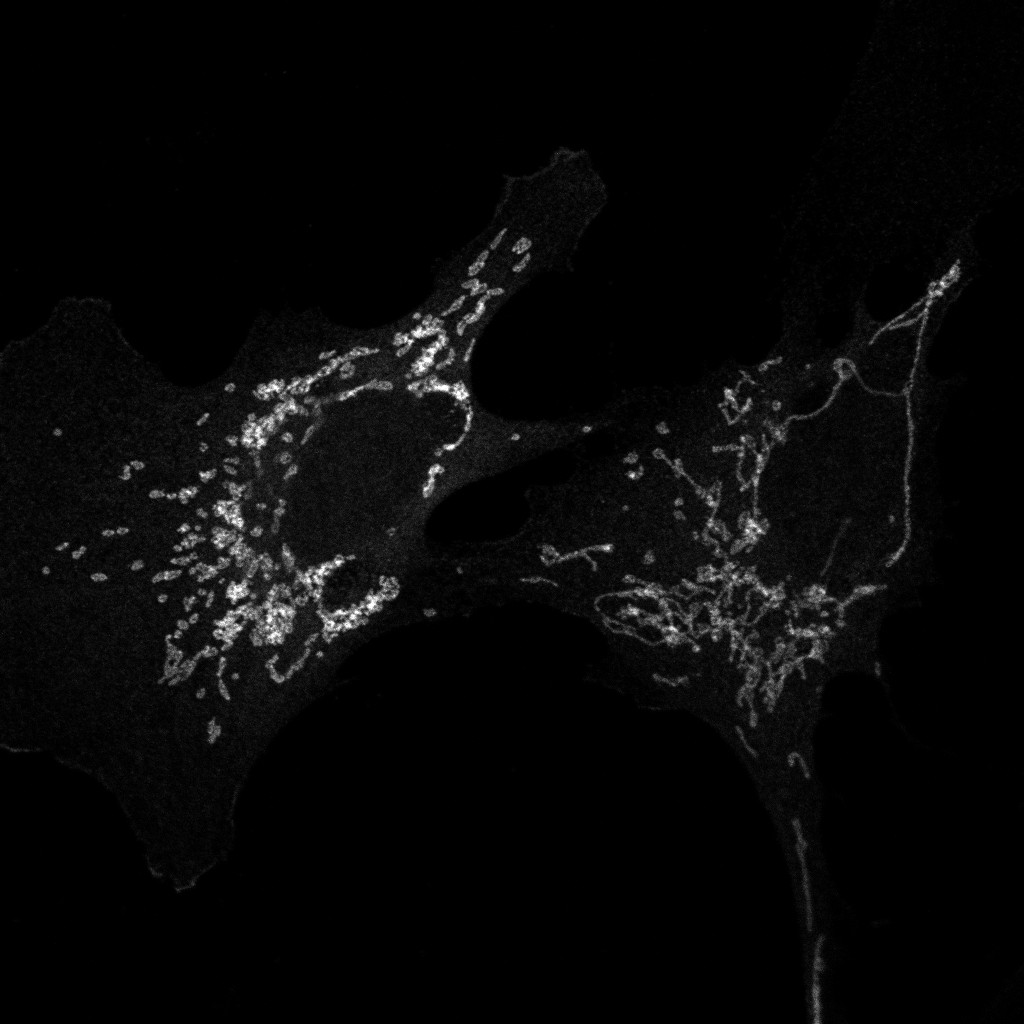

Supplement: Supplementary file 8 — Source Data for Figure 4 [file EMBJ-42-e112799-s003.zip › EMBOJ-2022-112799R_Figure4/Figure 4G/KO2_WT-FLAG.tif]

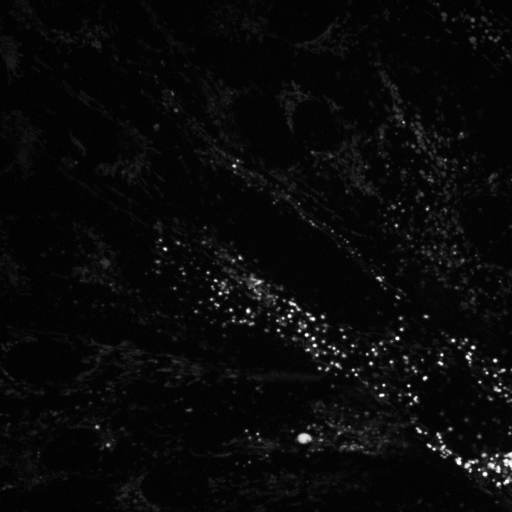

Supplement: Supplementary file 9 — Source Data for Figure 5 [file EMBJ-42-e112799-s006.zip › EMBOJ-2022-112799R_Figure5/Figure 5A/HEPD-Exp159-KO2-untreated - Ex.561.tif]

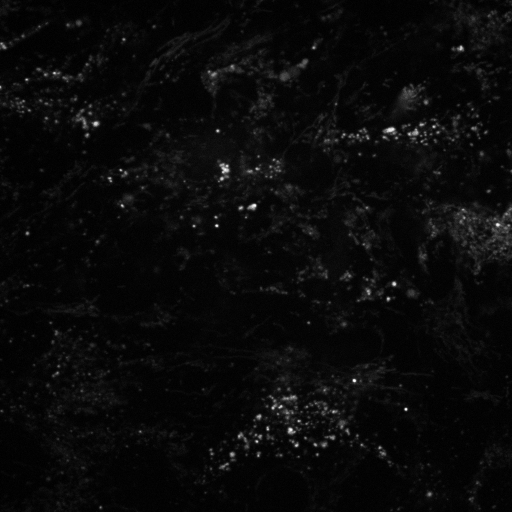

Supplement: Supplementary file 9 — Source Data for Figure 5 [file EMBJ-42-e112799-s006.zip › EMBOJ-2022-112799R_Figure5/Figure 5A/HEPD-Exp159-KO2-NT1 - Ex.561.tif]

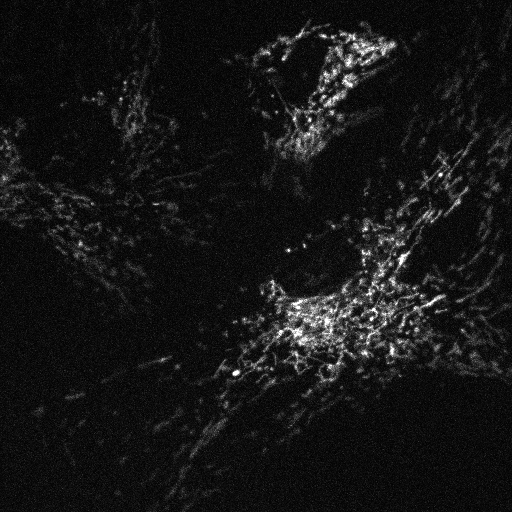

Supplement: Supplementary file 9 — Source Data for Figure 5 [file EMBJ-42-e112799-s006.zip › EMBOJ-2022-112799R_Figure5/Figure 5A/HEPD-Exp159-KO2-HIF1 - Ex.455.tif]

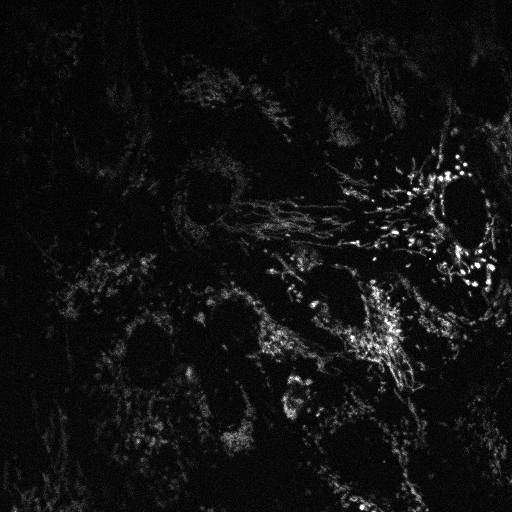

Supplement: Supplementary file 9 — Source Data for Figure 5 [file EMBJ-42-e112799-s006.zip › EMBOJ-2022-112799R_Figure5/Figure 5A/HEPD-Exp159-Control-untreated - Ex.455.tif]

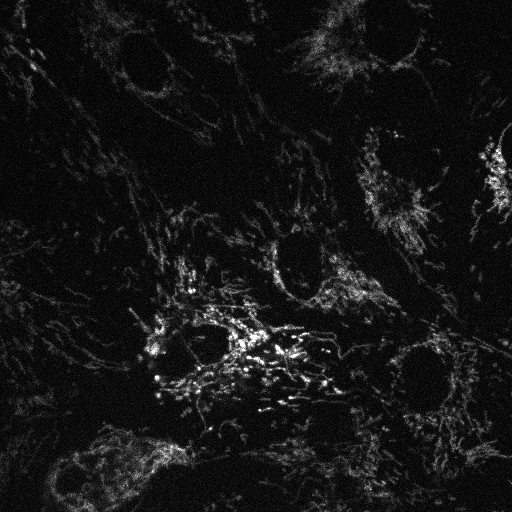

Supplement: Supplementary file 9 — Source Data for Figure 5 [file EMBJ-42-e112799-s006.zip › EMBOJ-2022-112799R_Figure5/Figure 5A/HEPD-Exp159-KO2-Nix - Ex.455.tif]

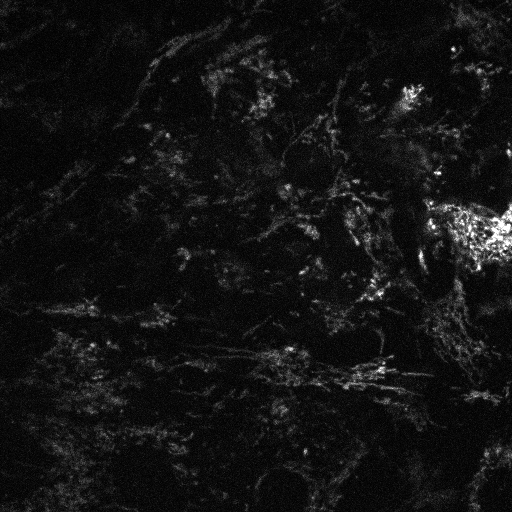

Supplement: Supplementary file 9 — Source Data for Figure 5 [file EMBJ-42-e112799-s006.zip › EMBOJ-2022-112799R_Figure5/Figure 5A/HEPD-Exp159-KO2-NT1 - Ex.455.tif]

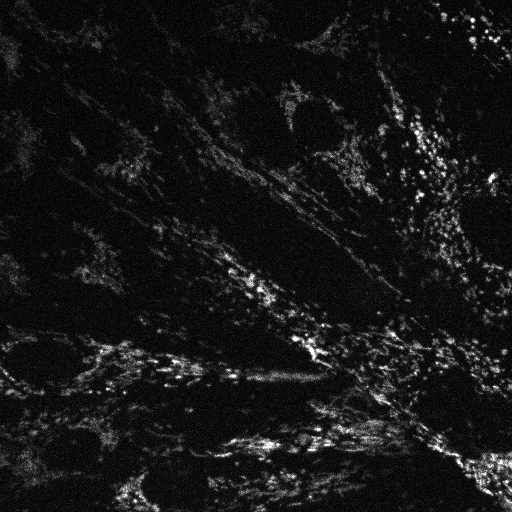

Supplement: Supplementary file 9 — Source Data for Figure 5 [file EMBJ-42-e112799-s006.zip › EMBOJ-2022-112799R_Figure5/Figure 5A/HEPD-Exp159-KO2-untreated - Ex.455.tif]

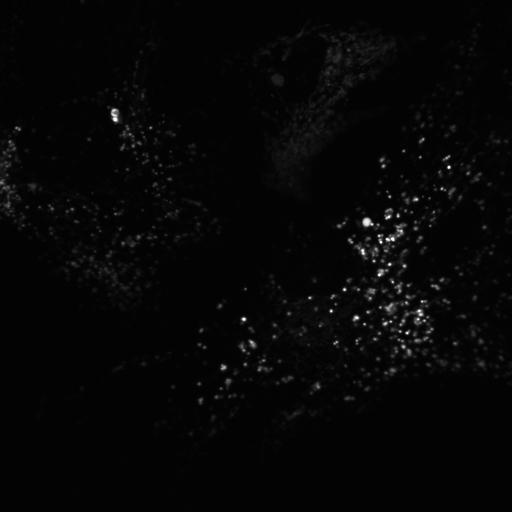

Supplement: Supplementary file 9 — Source Data for Figure 5 [file EMBJ-42-e112799-s006.zip › EMBOJ-2022-112799R_Figure5/Figure 5A/HEPD-Exp159-KO2-HIF1 - Ex.561.tif]

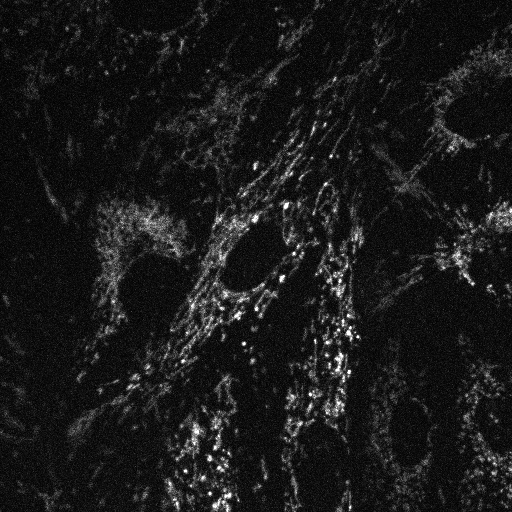

Supplement: Supplementary file 9 — Source Data for Figure 5 [file EMBJ-42-e112799-s006.zip › EMBOJ-2022-112799R_Figure5/Figure 5A/HEPD-Exp159-KO2-BNIP3 -Ex.455.tif]

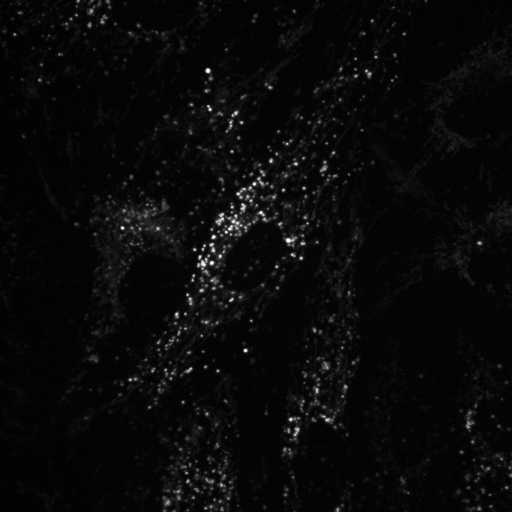

Supplement: Supplementary file 9 — Source Data for Figure 5 [file EMBJ-42-e112799-s006.zip › EMBOJ-2022-112799R_Figure5/Figure 5A/HEPD-Exp159-KO2-BNIP3 - Ex.561.tif]

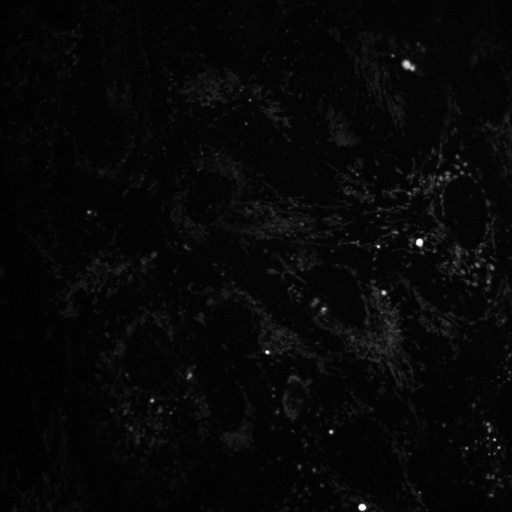

Supplement: Supplementary file 9 — Source Data for Figure 5 [file EMBJ-42-e112799-s006.zip › EMBOJ-2022-112799R_Figure5/Figure 5A/HEPD-Exp159-Control-untreated - Ex.561.tif]

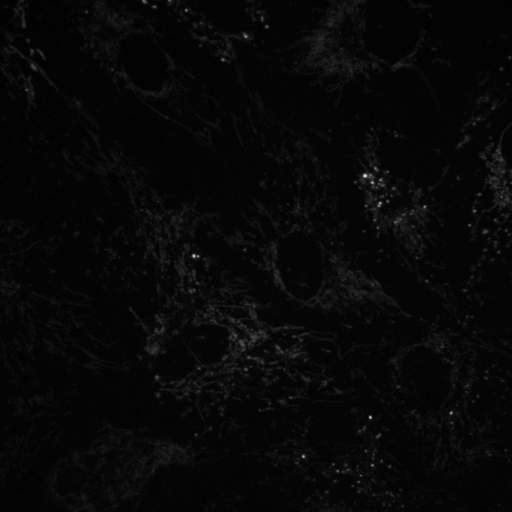

Supplement: Supplementary file 9 — Source Data for Figure 5 [file EMBJ-42-e112799-s006.zip › EMBOJ-2022-112799R_Figure5/Figure 5A/HEPD-Exp159-KO2-Nix - Ex.561.tif]

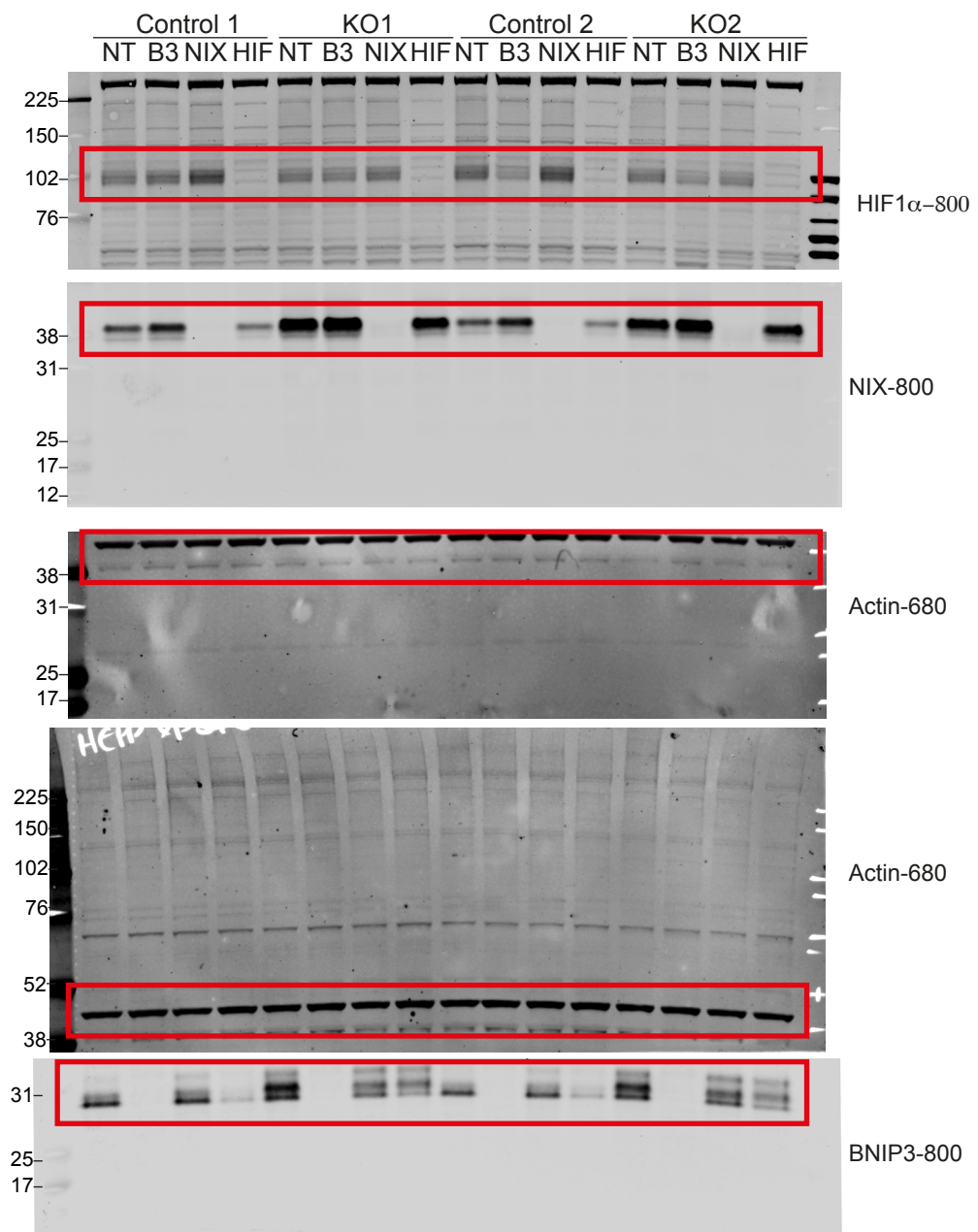

Supplement: Supplementary file 9 — Source Data for Figure 5 [file EMBJ-42-e112799-s006.zip › EMBOJ-2022-112799R_Figure5/Figure 5C/Figure 5C-western blot.pdf]

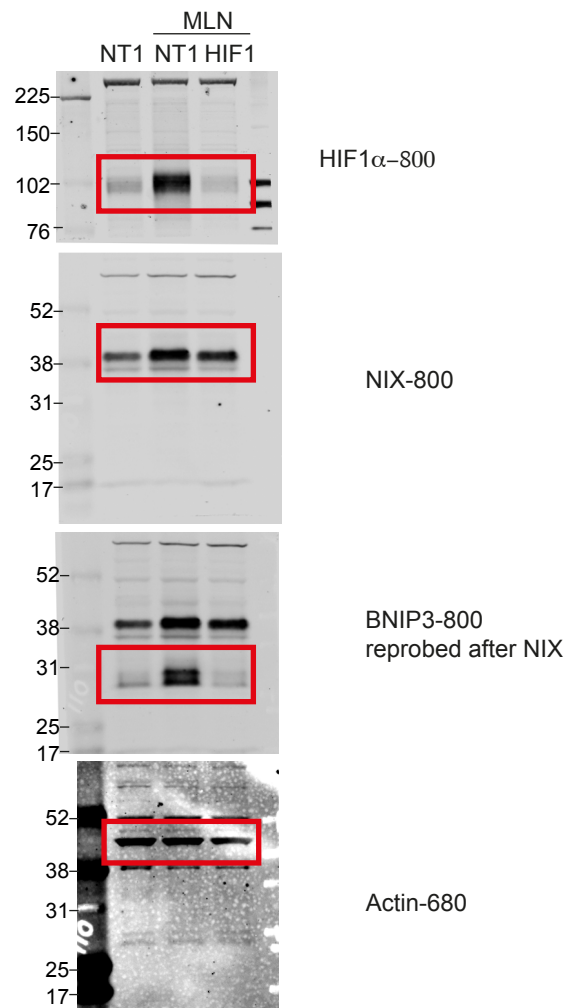

Supplement: Supplementary file 10 — Source Data for Figure 6 [file EMBJ-42-e112799-s008.zip › EMBOJ-2022-112799R_Figure6/Figure 6F/Figure 6F-Western Blot.pdf]

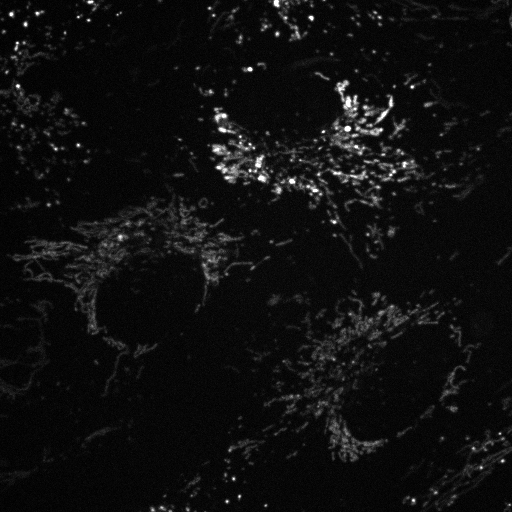

Supplement: Supplementary file 10 — Source Data for Figure 6 [file EMBJ-42-e112799-s008.zip › EMBOJ-2022-112799R_Figure6/Figure 6A/DMSO-Ex.561.tif]

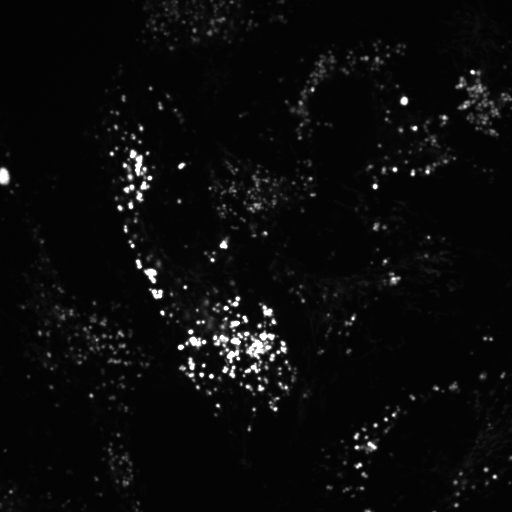

Supplement: Supplementary file 10 — Source Data for Figure 6 [file EMBJ-42-e112799-s008.zip › EMBOJ-2022-112799R_Figure6/Figure 6A/MLN-Ex.561.tif]

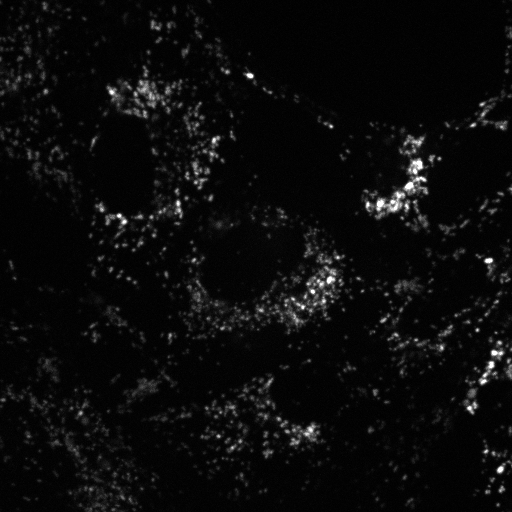

Supplement: Supplementary file 10 — Source Data for Figure 6 [file EMBJ-42-e112799-s008.zip › EMBOJ-2022-112799R_Figure6/Figure 6A/AO-Ex.455.tif]

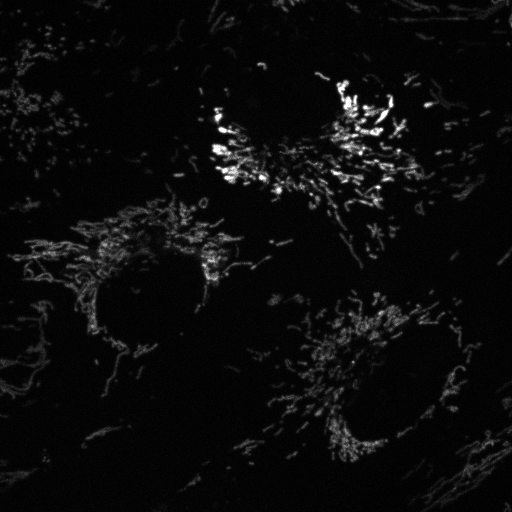

Supplement: Supplementary file 10 — Source Data for Figure 6 [file EMBJ-42-e112799-s008.zip › EMBOJ-2022-112799R_Figure6/Figure 6A/DMSO-Ex.455.tif]

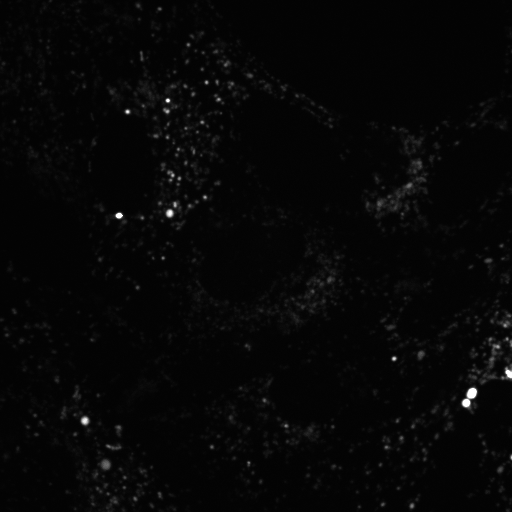

Supplement: Supplementary file 10 — Source Data for Figure 6 [file EMBJ-42-e112799-s008.zip › EMBOJ-2022-112799R_Figure6/Figure 6A/AO-Ex.561.tif]

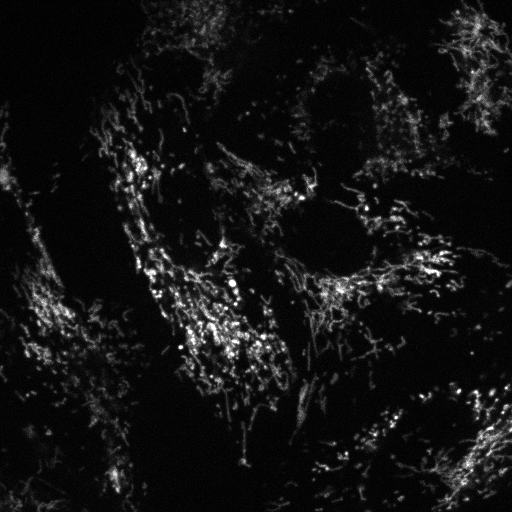

Supplement: Supplementary file 10 — Source Data for Figure 6 [file EMBJ-42-e112799-s008.zip › EMBOJ-2022-112799R_Figure6/Figure 6A/MLN-Ex.455.tif]

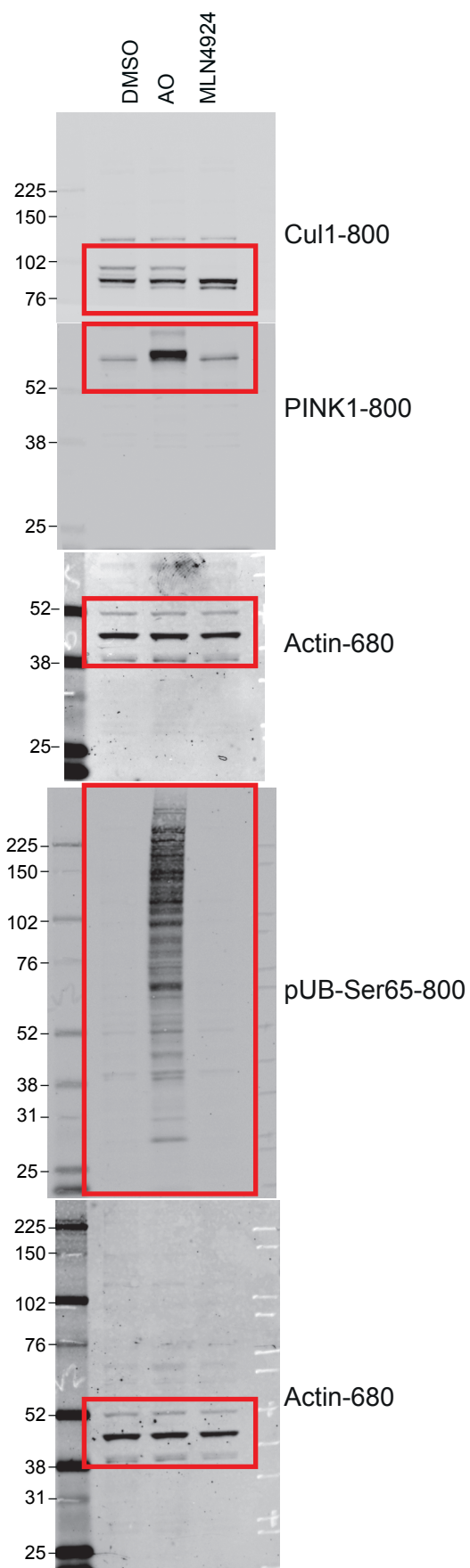

Supplement: Supplementary file 10 — Source Data for Figure 6 [file EMBJ-42-e112799-s008.zip › EMBOJ-2022-112799R_Figure6/Figure 6B/Figure 6B-Western blot.pdf]

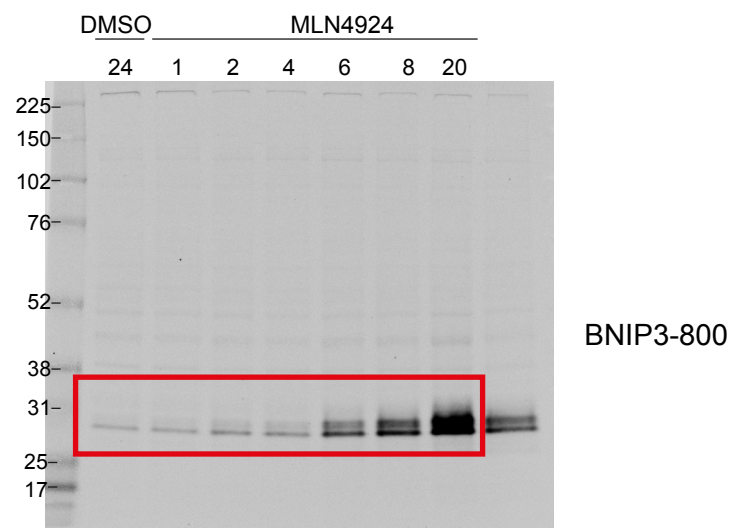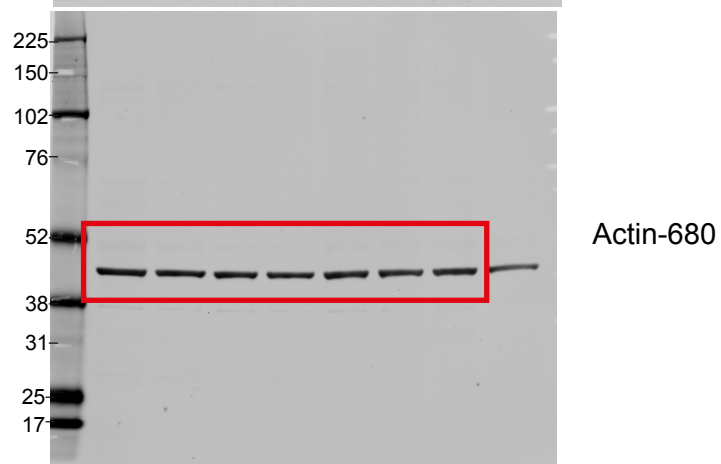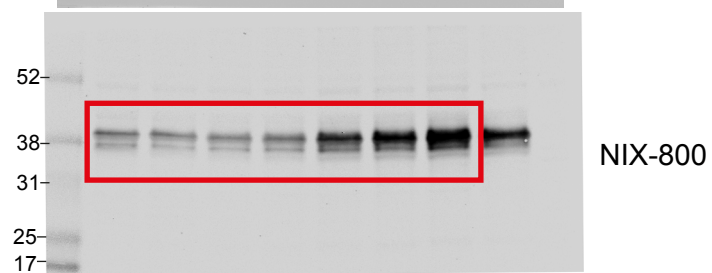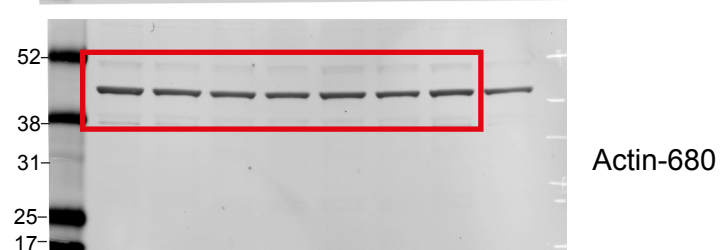

Supplement: Supplementary file 10 — Source Data for Figure 6 [file EMBJ-42-e112799-s008.zip › EMBOJ-2022-112799R_Figure6/Figure 6E/Figure 6E-Western blot.pdf]

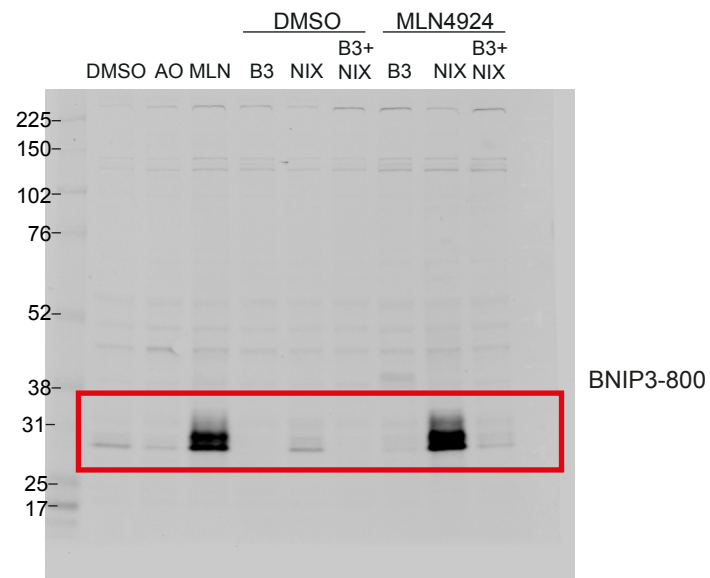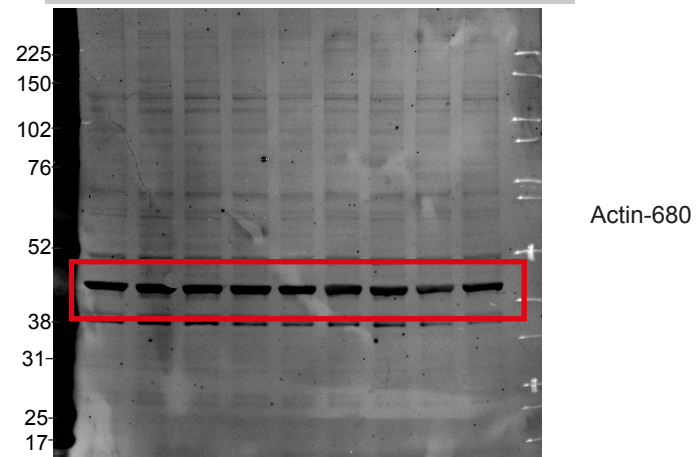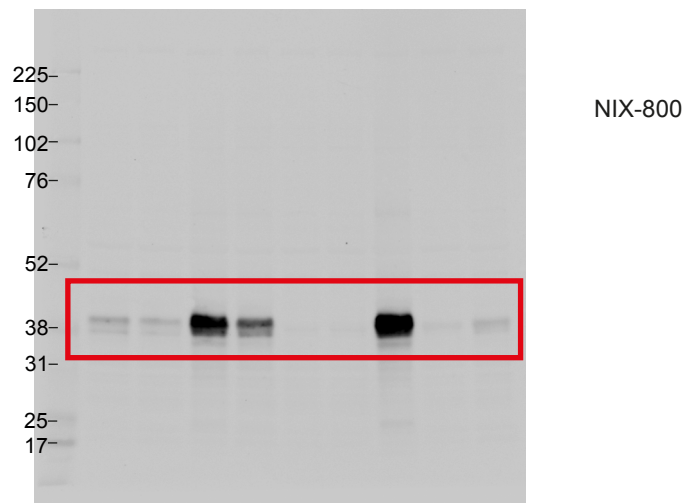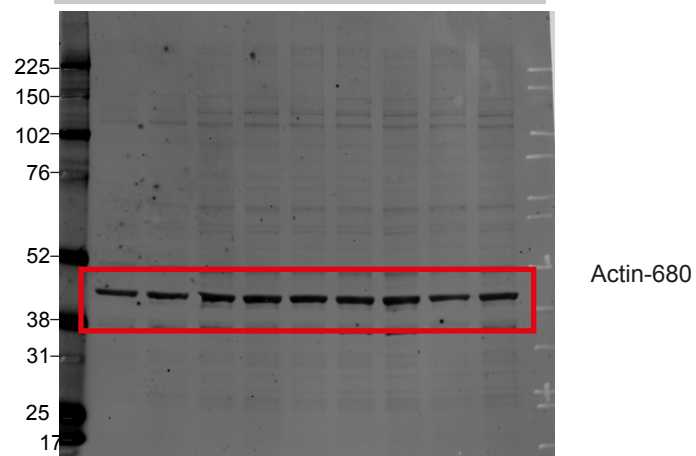

Supplement: Supplementary file 10 — Source Data for Figure 6 [file EMBJ-42-e112799-s008.zip › EMBOJ-2022-112799R_Figure6/Figure 6C/Figure 6C western blot.pdf]

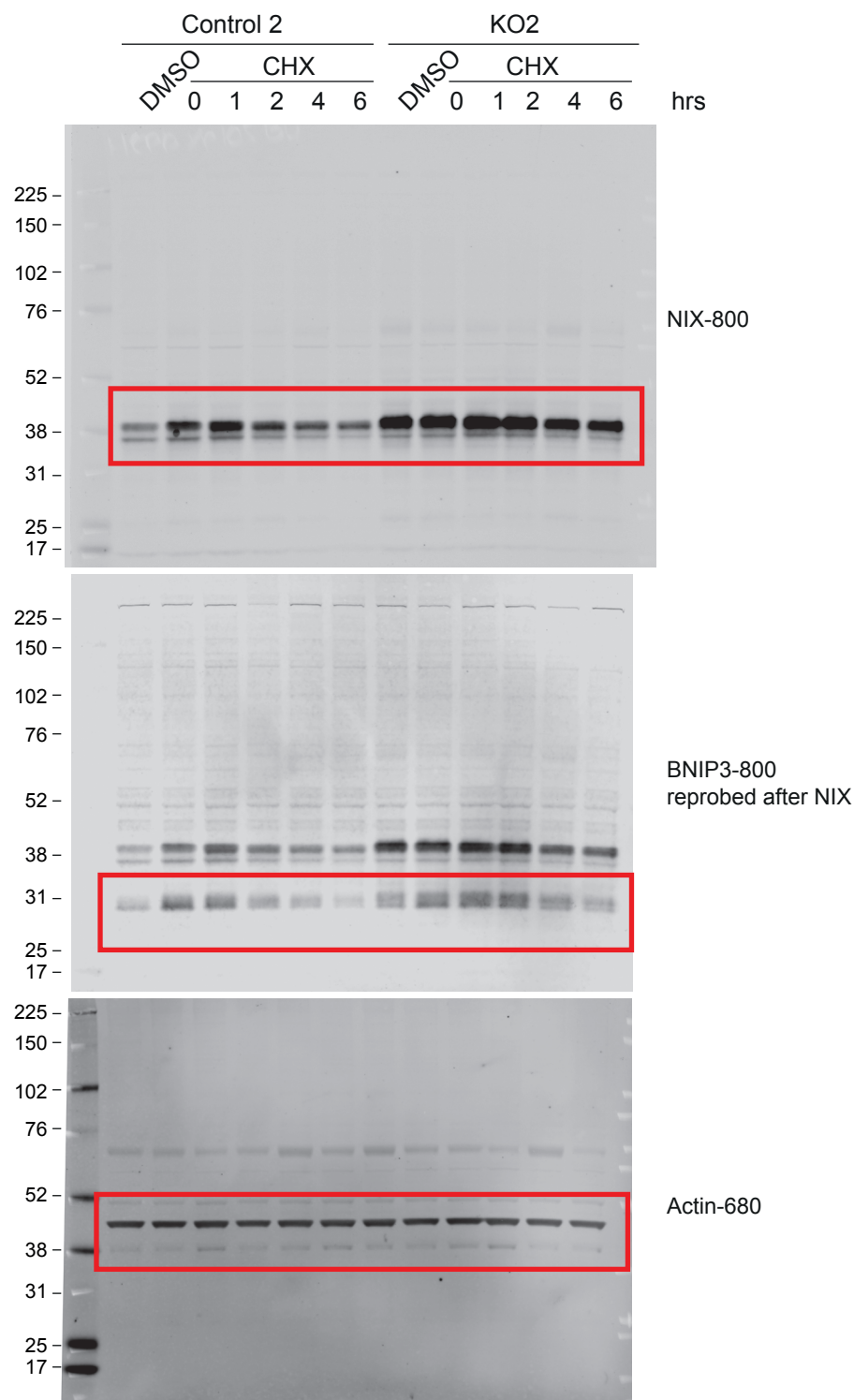

Supplement: Supplementary file 11 — Source Data for Figure 7 [file EMBJ-42-e112799-s002.zip › EMBOJ-2022-112799R_Figure7/Figure 7B/Figure 7B-western blot .pdf]

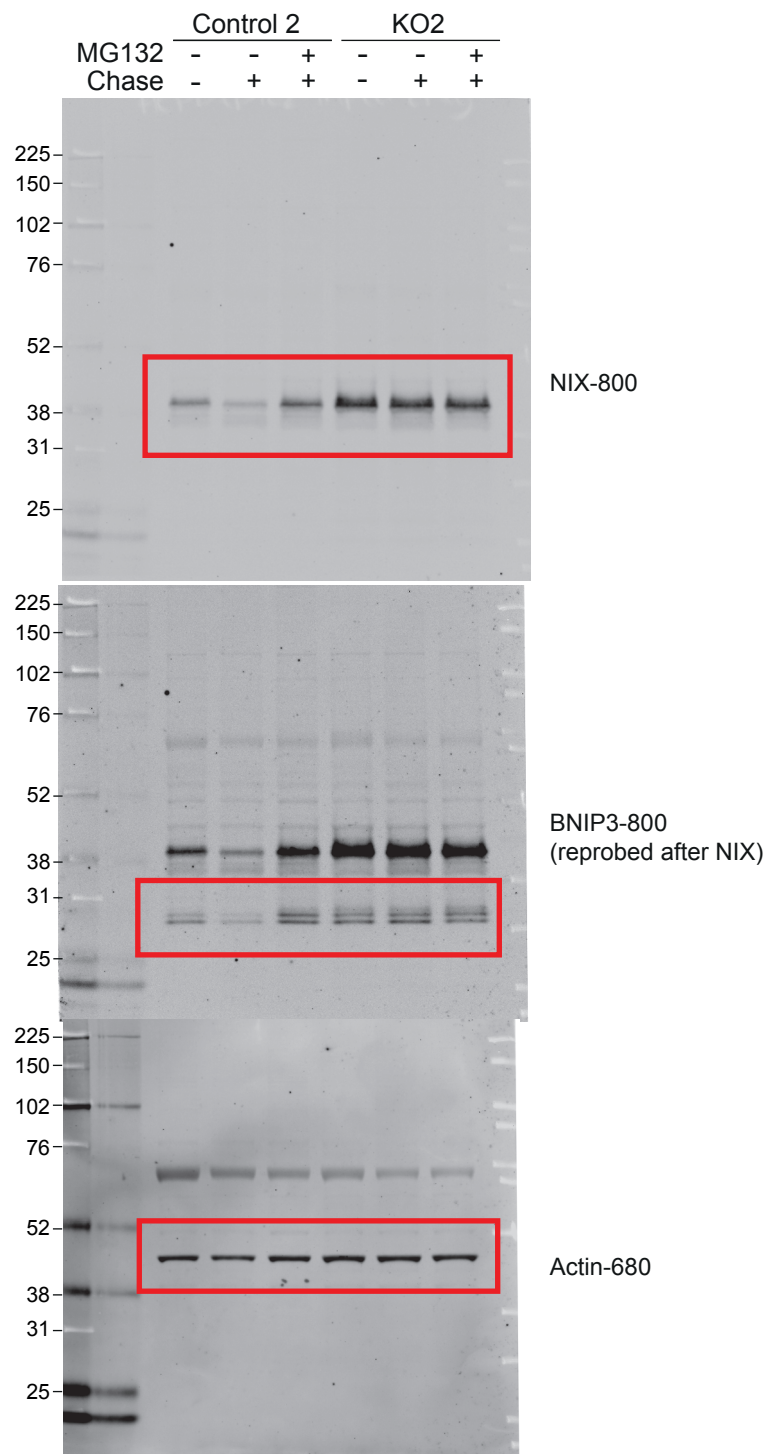

Supplement: Supplementary file 11 — Source Data for Figure 7 [file EMBJ-42-e112799-s002.zip › EMBOJ-2022-112799R_Figure7/Figure 7E/EMBOJ-2022-112799R-7E_Source_Data-sd.pdf]

|       | Ctrl2    |   |   |    | KO2      |   |   |    |
|-------|----------|---|---|----|----------|---|---|----|
|       | TUBES PD |   |   | CB | TUBES PD |   |   | CB |
| MG132 | -        | - | + | +  | -        | - | + | +  |
| Chase | -        | + | + | +  | -        | + | + | +  |

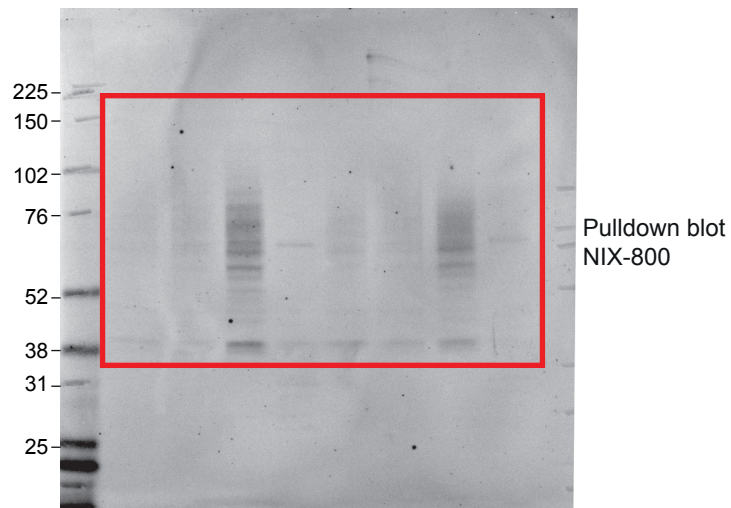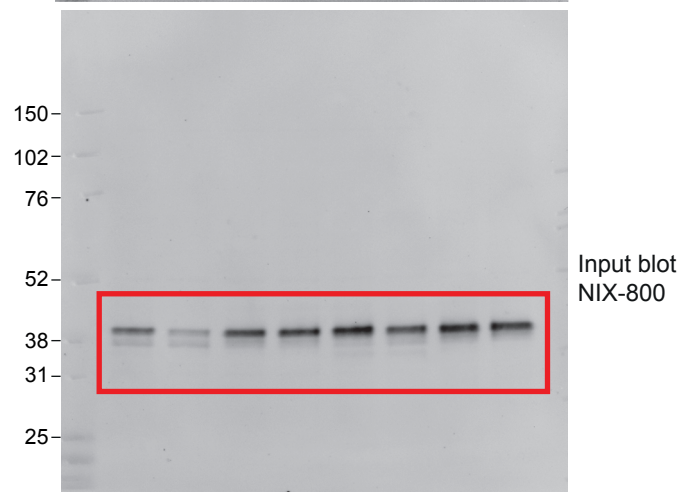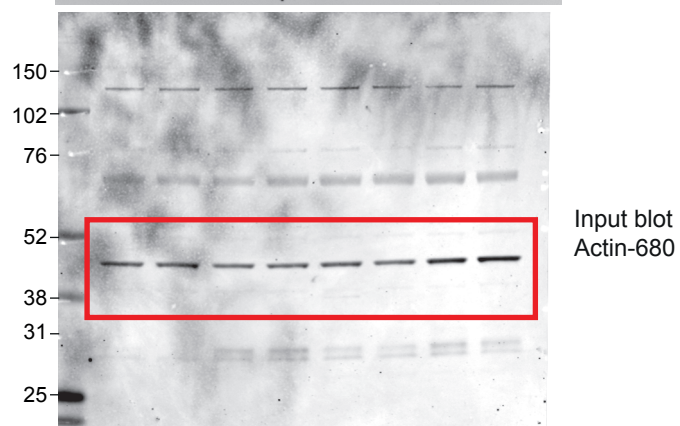

Supplement: Supplementary file 11 — Source Data for Figure 7 [file EMBJ-42-e112799-s002.zip › EMBOJ-2022-112799R_Figure7/Figure 7F/Figure 7F-Western blot.pdf]

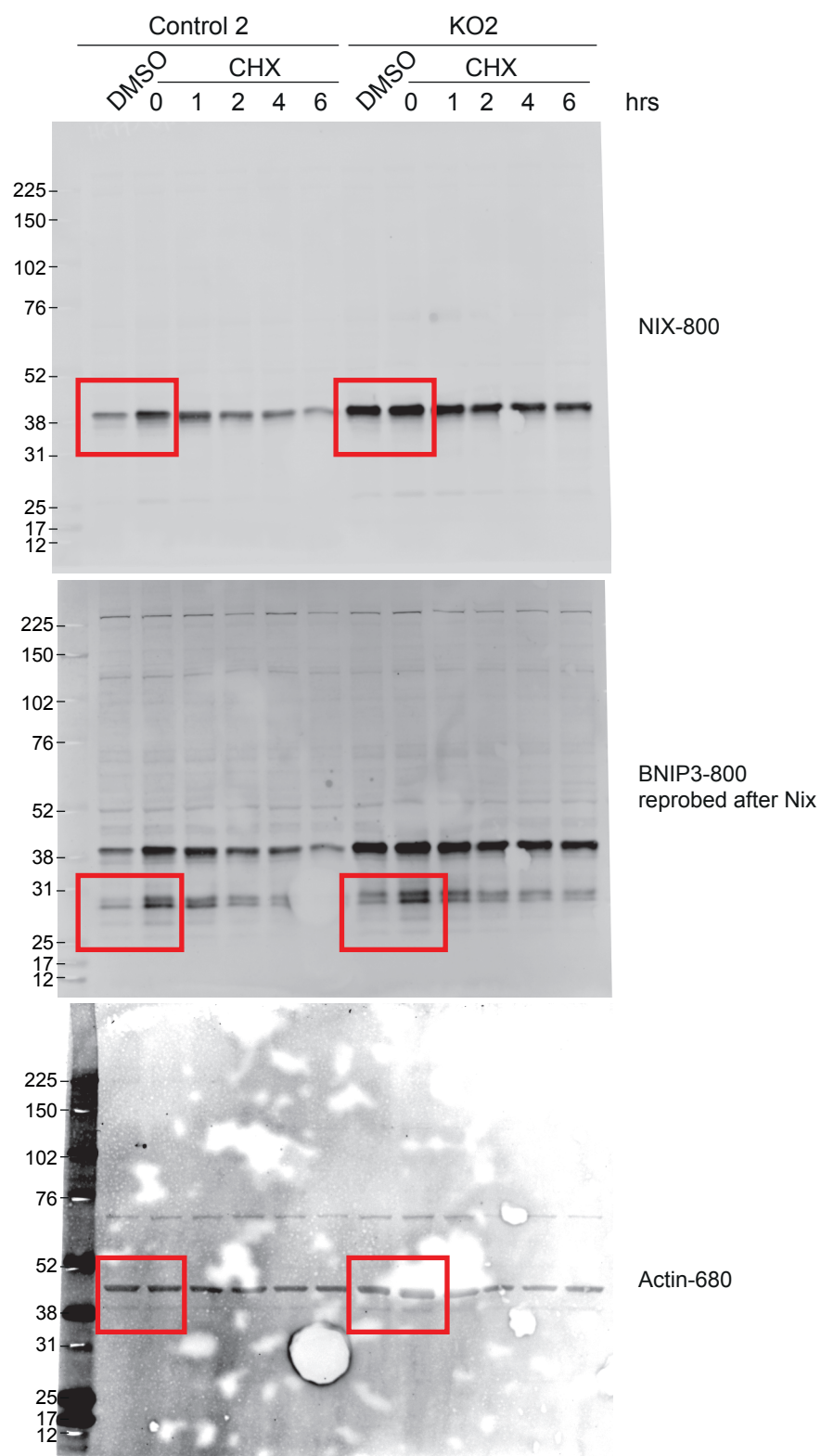

Supplement: Supplementary file 11 — Source Data for Figure 7 [file EMBJ-42-e112799-s002.zip › EMBOJ-2022-112799R_Figure7/Figure 7A/Figure 7A-Western blot.pdf]
