# Supplementary material for: FBXL4 ubiquitin ligase deficiency promotes mitophagy by elevating NIX levels
Source: EMBO J. 2023 Apr 27;42(13):e112799. doi: 10.15252/embj.2022112799 (PMC10308357; doi:10.15252/embj.2022112799)
Supplement: Supplementary file 12 [file EMBJ-42-e112799-s005.pdf]

Expanded View Figures

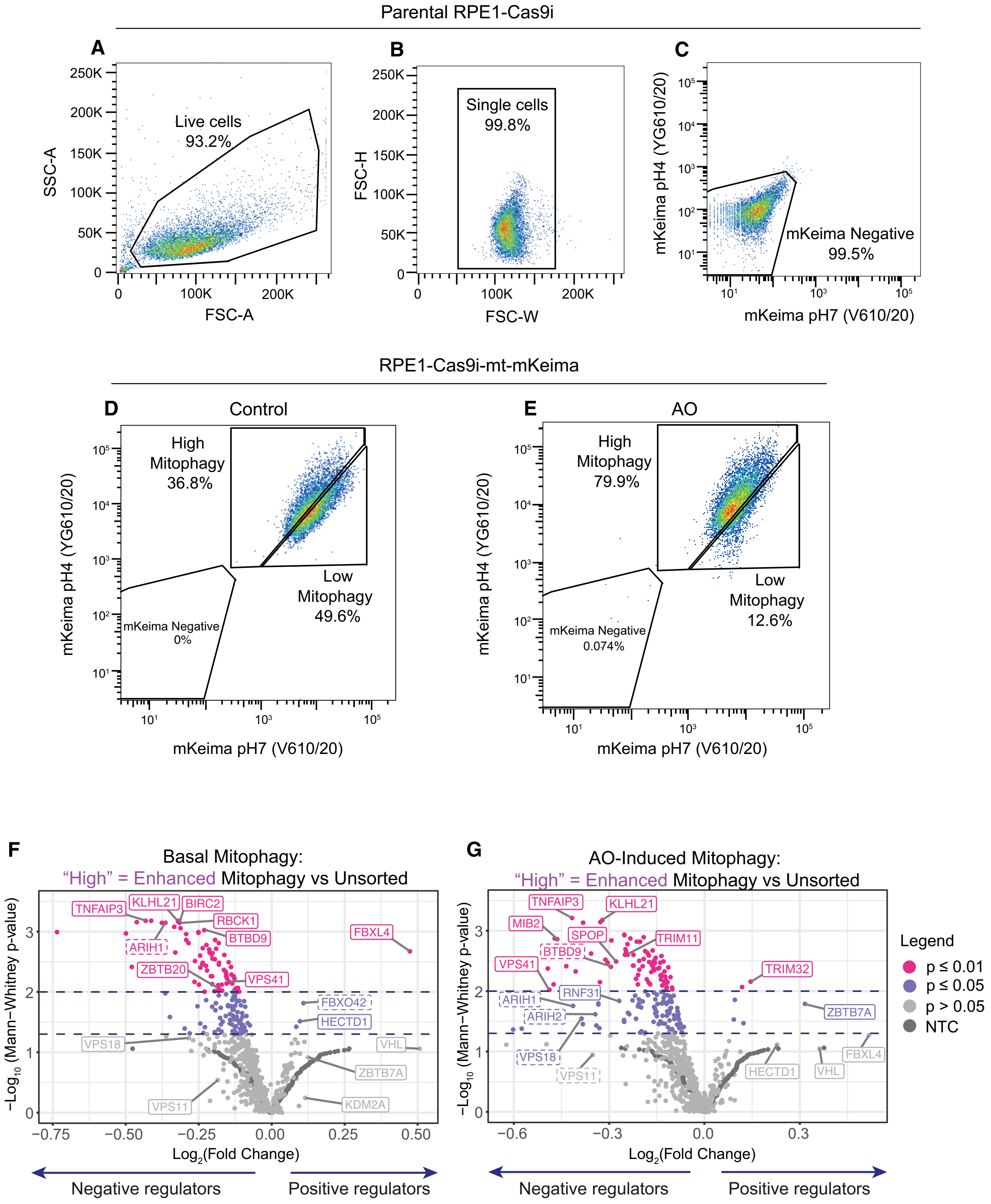

Figure EV1.

© 2023 The Authors

Figure EV1. Characterization of RPE1-Cas9i cells stably expressing mt-mKeima.

A–C Parental RPE1-Cas9i cells were used to set the gates for flow cytometry for live (A) and single cells (B) and to exclude non-fluorescent cells (C).

D, E RPE1-Cas9i-mt-mKeima cells were treated with DMSO (D) or antimycin A and oligomycin A (AO; 1 and 10 μM, respectively) (E) for 24 h. An increase in the proportion of cells undergoing mitophagy (high mitophagy population) is observed in AO-treated cells.

F, G Volcano plots showing the average Log_2 fold change and $-\text{Log}_{10}$ *P*-value of genes in high mitophagy versus unsorted cells in the induced and basal mitophagy screen for two independent biological replicates. Statistical thresholds of 2 and 3 standard deviations from the mean are indicated by dashed lines and colour coding. Indicated are high-confidence (unbroken line) and lower-confidence (dashed line) candidates shown in Fig 1D–F.

Figure EV2. Supplementary data for Figs 1–3.

A Quantitative RT–PCR reactions of FBXL4 (normalized to actin) were performed with cDNA derived from hTERT-RPE1 cells transfected with non-targeting (NT1) or siRNA-targeting FBXL4. Two primer pairs were used and are indicated by symbol shape. Shown is the fold change normalized to NT1 control samples for three independent colour-coded experiments. Error bars show standard deviation; unpaired *t*-test, ****$P < 0.0001$.

B Quantitative RT–PCR reactions of BNIP3 or NIX (normalized to actin) were performed with cDNA derived from hTERT-RPE1 cells transfected with non-targeting (NT1) or siRNA-targeting FBXL4 or HIF1α (HIF). Two primer pairs were used per target indicated by different symbol shapes. Shown is the fold change normalized to the NT1 control samples for three independent colour-coded experiments. Error bars show standard deviation; one-way ANOVA and Dunnett's multiple-comparison test, **$P < 0.01$, ***$P < 0.001$ and ****$P < 0.0001$.

C Sequencing analysis of FBXL4-knockout RPE1-Cas9i-mt-mkeima cell pools KO1 and KO2 using Synthego ICE analysis. Shown are the predicted edit and KO efficiency for each pool.

D Western blot of lysates from matched control (Ctrl) and FBXL4 knockout RPE1-Cas9i-mt-mkeima cell pools probed for the mitochondrial protein TOMM40.

E Quantification of data shown in D for four colour-coded independent experiments. Error bars show standard deviation. One-way ANOVA with Tukey's multiple-comparison test, *$P < 0.05$.

F Western blot of lysates as in (D) probed for cathepsin D. Bands corresponding to mature and pro-cathepsin D (Pro) are indicated. A ponceau stain is shown to illustrate equal loading across the length of the lane. *Indicates a non-specific band.

Source data are available online for this figure.

© 2023 The Authors

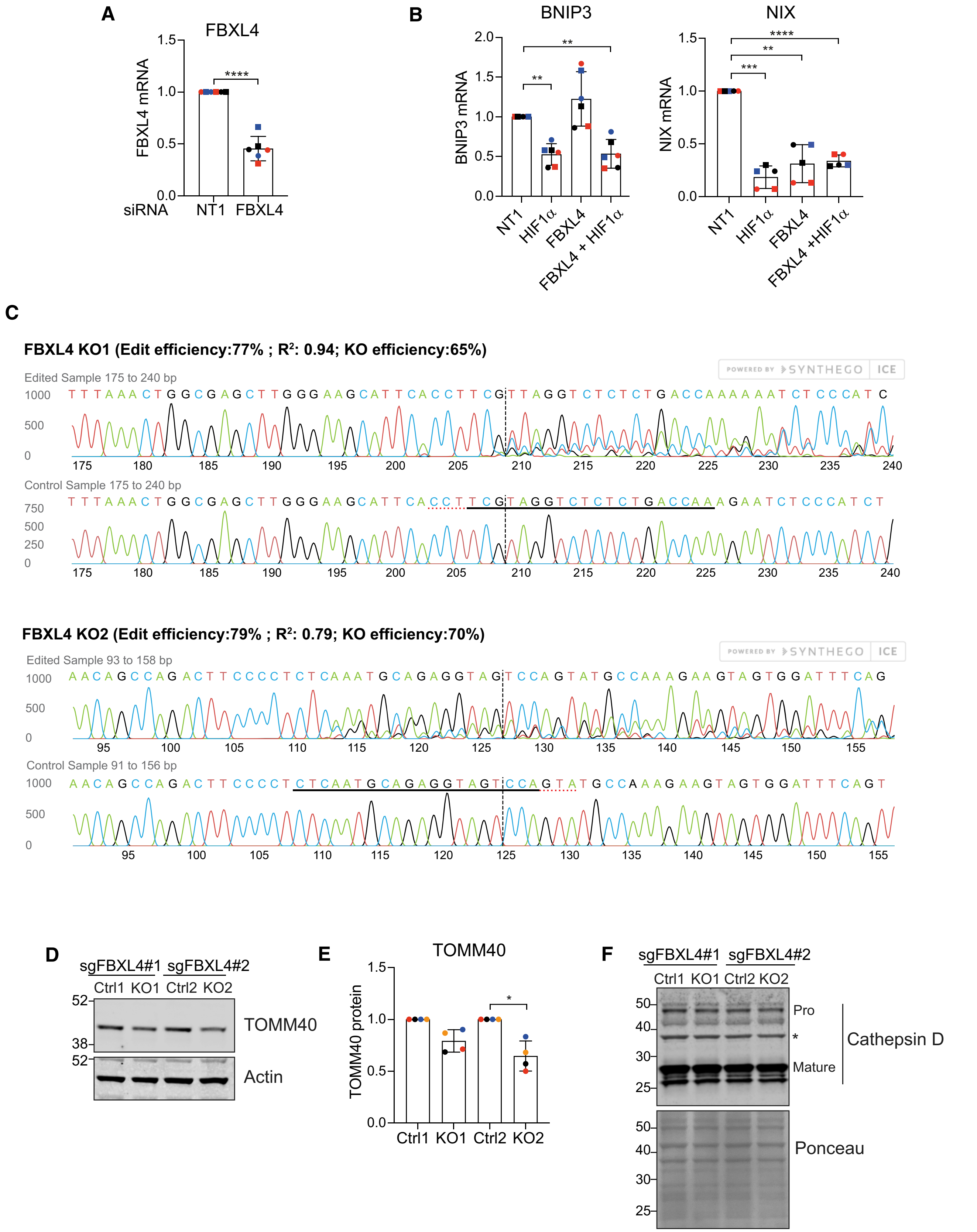

Figure EV2.

© 2023 The Authors

Figure EV3. Accessory data indicating FBXL4 influences NIX and BNIP3 stability.

A Representative western blot of hTERT-RPE1 cells treated with DMSO, antimycin (1 μM) and oligomycin (10 μM) (AO) or MLN4924 (1 μM) for 24 h.

B Representative images of hTERT-RPE1 cells treated with DMSO or MLN4924 (1 μM) for 24 h, fixed and stained for either BNIP3 or NIX and for TOMM20. Scale bar 20 μm.

C Quantitative RT–PCR reactions of BNIP3 or NIX (normalized to Actin) were performed with cDNA derived from hTERT-RPE1 cells treated with DMSO or MLN4924 (1 μM) for 24 h. Two primer pairs were used per target indicated by different symbol shapes. Shown is the fold change normalized to the DMSO control sample for three independent colour-coded experiments. Error bars show standard deviation; unpaired *t*-test, $^{***}P < 0.001$ and $^{****}P < 0.0001$.

D Representative western blot of hTERT-RPE1 cells pre-treated with DMSO or MLN4924 (1 μM) for 6 h, followed by a cycloheximide chase (100 μg/ml).

E Representative western blot of lysates from control (Ctrl1) or FBXL4 KO1 RPE1-Cas9i-mt-mKeima cells pre-treated without or with MLN4924 (1 μM) for 6 h followed by a cycloheximide (CHX) chase (100 μg/ml).

F, G Quantification of protein levels calculated from data represented in (E) for three independent colour-coded experiments. Error bars show standard deviation.

H Representative western blot of hTERT-RPE1 cells transfected for 72 h with siRNA against indicated targets or non-targeting (NT1) prior to harvesting and cell lysis.

Source data are available online for this figure.

© 2023 The Authors

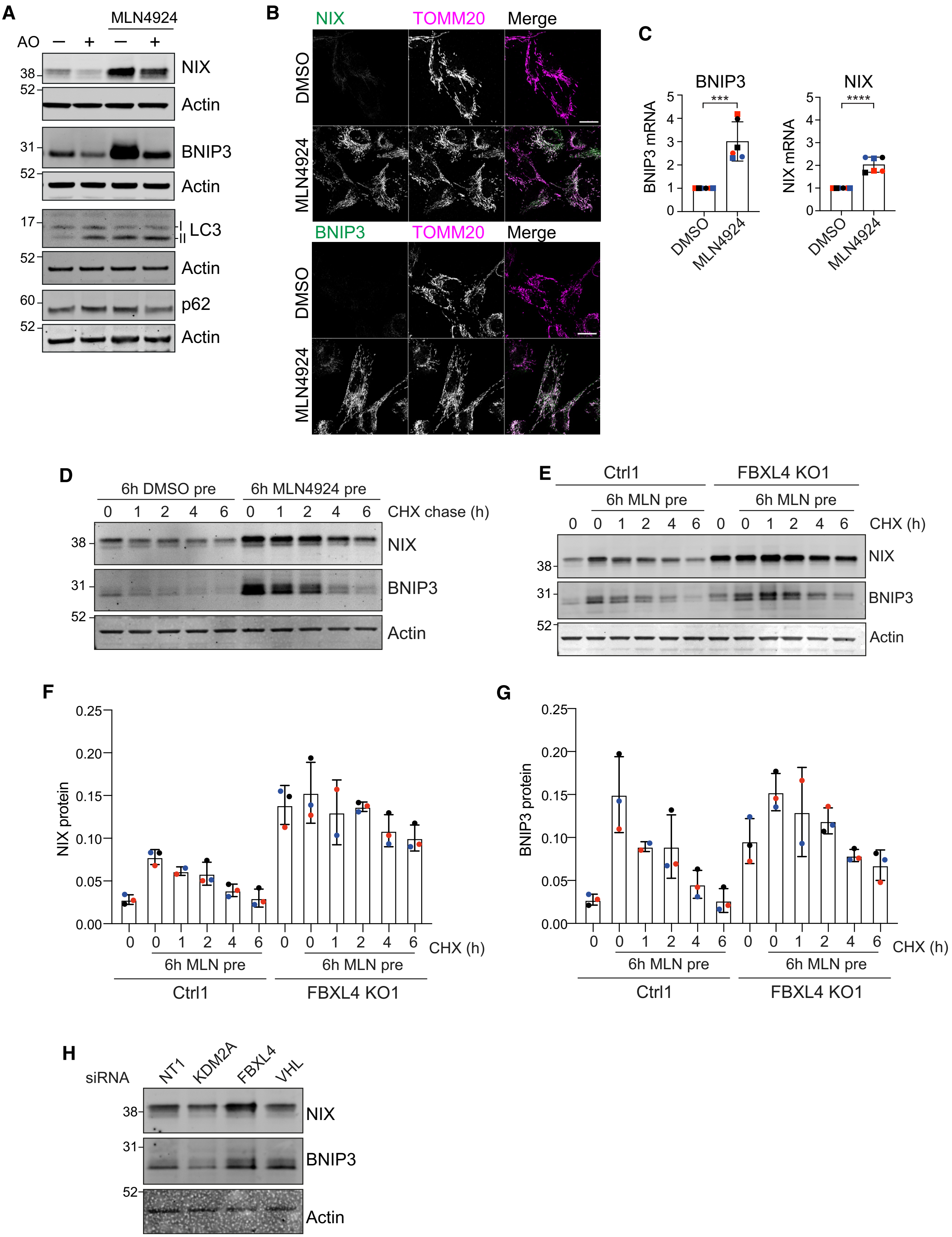

Figure EV3.

© 2023 The Authors
